# AWaRe classification analysis for European countries with ARIMA forecasts to assess prescribing patterns and ‘One Health’ targets

**DOI:** 10.1007/s00210-025-04121-y

**Published:** 2025-04-12

**Authors:** Lilly Josephine Bindel, Roland Seifert

**Affiliations:** Institute of Pharmacology Hannover Medical School, 30625 Hannover, Germany

**Keywords:** Antimicrobial consumption, Antibiotic, Antibacterial drug, AMR, AMC, Antibiotic prescription, Europe, Surveillance, Antibiotic Stewardship, Rational prescribing behaviour, Irrational prescribing behaviour, Forecast, ARIMA, One Health, ECDC, EU, AWaRe

## Abstract

**Background:**

Antimicrobial resistance is a major threat to public health, with a well-established link between antibacterial consumption and bacterial resistance. Stewardship needs to focus on reducing overall consumption and optimising the quality of prescribing. The European Union’s ‘One Health’ approach aims for at least 65% of antibacterial consumption to be in the ‘Access’ category of the AWaRe framework until 2030.

**Purpose:**

This study advances the field by shifting the focus from simply quantifying antibacterial consumption to a nuanced assessment of prescribing quality. Prudent and problematic prescribing behaviour is identified in past and projected trends, both for individual countries and European regions. Progress towards the EU’s ‘One Health’ targets for the human sector is assessed and compared with total antibacterial consumption.

**Methods:**

This study analyses data from 1997–2023 and predicts future proportions of AWaRe drug classes for European countries until 2030, using the ARIMA(1,0,1) model. The distribution and changes of the AWaRe groups were analysed to assess prescribing behaviour. Total antibacterial consumption is compared with findings of the ‘One Health’ targets, and bivariate correlation analyses are performed.

**Results:**

Robust forecast models were developed for the AWaRe classification for 30 European countries. In 2030, the ‘Access’ group is projected to exceed 65% in Iceland, Denmark, Ireland, Latvia, Finland, France, the Netherlands, Sweden, Lithuania, the United Kingdom, Belgium and Estonia. On the other hand, low shares are expected for Greece (43.8%), Slovakia and Bulgaria (45.9%), Italy (47.3%), Malta (49.9%), Cyprus (50.9%), Hungary (51.8%) and Romania (53.4%). The other 10 countries fall in between, with shares ranging from 56.4% to 64.7%. Of particular concern are Italy, Cyprus and Malta, with low'Access'shares, high consumptions and worsening trends, in contrast to the exemplary performance of Iceland, Norway and Denmark. Germany stands out for its worryingly excessive use of ‘Reserve’. Most forecasts were considered reliable, although some showed moderate or poor fit.

**Conclusion:**

The findings predict that many European countries are unlikely to meet the EU's ‘One Health’ target by 2030. Countries with higher'Access'shares and lower total consumption tend to have lower levels of bacterial resistance, while those with high consumption and problematic prescribing patterns face higher levels of resistance. While most Northern European countries are considered to have a prudent use of medicines, problematic use is observed particularly in Southern and Eastern Europe, a practice being apparent across many medicine classes. Regional differences in prescribing patterns highlight the need for tailored interventions. For certain countries, particularly in Northern Europe, the high proportion of unclassified substances suggests that the AWaRe classification may not fully capture the range of antibacterial substances used.

**Supplementary Information:**

The online version contains supplementary material available at 10.1007/s00210-025-04121-y.

## Introduction

“Antimicrobial resistance (AMR) remains a major public threat, with antibiotic-resistant infections occurring in the EU resulting in approximately 35´000 deaths every year and directs costs estimated at EUR 6.6 billion” (OECD 2024). These alarming consequences underscore the critical link between antibacterial consumption and bacterial resistance (Abejew et al. 2024; Olesen et al. 2018), highlighting the urgent need “for improvements in antimicrobial stewardship to optimise antibiotic use” (OECD 2024).

The WHO's AWaRe framework serves as a tool to assess the quantitative use of antibacterial drugs, being categorized in the groups ‘Access’, ‘Watch’ and ‘Reserve’. The distribution of the AWaRe groups helps to identify prudent and problematic prescribing behaviour. Further information about the classification criteria, characteristics and examples of listed substances can be found in Table 1. European action plans, such as the'One Health'approach of the EU (EU 2023), have introduced various measures aimed at reducing the consumption of antibacterial drugs by − 20%, promoting rational prescribing practices with at least 65% of the ‘Access’ group by the WHO's AWaRe framework, and to reduce bloodstream infections caused by resistant bacterial strains (EU 2023). Despite these efforts, progress has been slow (ECDC 2024a), and a more comprehensive analysis of consumption patterns and their impact on future AMR trends is needed (Bindel and Seifert 2025a, 2025b).

**Table 1:** Explanation of the AWaRe framework and its antibacterial drug groups ‘Access’, ‘Watch’, ‘Reserve’ and ‘Unclassified’. Beside a definition and characteristics, exemplary substances are listed. The AWaRe classification only includes antibacterial drugs, meaning that substance like antimycotics, antiparasitics or antivirals are excluded

**A**WaRe group	Definition (WHO 2023)	Characteristics (WHO 2023; Zanichelli et al. 2023)	Examples (WHO 2023)
'Access'	includes substances “that have activity against a wide range of commonly encountered susceptible pathogens while also showing lower resistance potential than […] other groups”	first‐ or second‐line options for common infections, comparable low resistance potential, should be widely available and affordable, primary role in community settings, preventing unnecessary escalation to broader-spectrum agents	Beta-lactams – Penicillin (Amoxicillin, Benzylpenicillin, Ampicillin) Beta-lactams – Cephalosporins 1 st Generation (Cephalexin, Cefadroxil) Beta-lactam/Beta-lactamase inhibitors (Amoxicillin clavulanic acid) Sulfonamides (Sulfamethoxazole-trimethoprim) Nitrofurans (Nitrofurantoin) Nitroimidazoles (Metronidazole) Tetracyclines (Doxycycline) Others (Chloramphenicol, Clindamycin, oral Fosfomycin)
'Watch'	includes substances"that have a higher resistance potential and include most of the highest priority agents"and"should be prioritised as key targets of stewardship programmes and surveillance", being"recommended as essential first or second choice empiric treatment options for a limited number of specific infectious syndromes"	associated with higher bacterial resistance risks, should be used cautiously, mainly used in hospitals, require antimicrobial stewardship to prevent resistance development	Cephalosporins 2nd, 3rd, 4 th Generation (Cefuroxime, Ceftriaxone, Cefotaxime, Ceftazidime, Cefepime) Beta-lactam/Beta-lactamase Inhibitors (Piperacillin/Tazobactam) Carbapenems (Imipenem, Meropenem, Ertapenem) Fluoroquinolones (Ciprofloxacin, Levofloxacin, Moxifloxacin) Macrolides (Azithromycin, Clarithromycin) Glycopeptides (Vancomycin)
'Reserve'	includes substances that"should be reserved for the treatment of confirmed or suspected infections caused by multidrug-resistant organisms"and"should be treated as options of last resort"	last-resort options, essential for treating infections caused by multidrug-resistant pathogens, strictly controlled use, limited to cases where all other treatments have failed, overuse poses a severe risk for resistance emergence	Polymyxins (Colistin, Polymyxin B) Advanced Beta-lactam/Beta-lactamase Inhibitors (Ceftazidime/Avibactam, Ceftolozane/Tazobactam) Glycylcyclines (Tigecycline) Fosfomycin IV (Intravenous Fosfomycin) Oxazolidinones (Linezolid) Lipopeptides (Daptomycin) Siderophore Cephalosporins (Cefiderocol)
'Unclassified'	“Other antibacterial drugs include those that are not part of the AWaRe classification but are essential for specific infectious diseases.” (WHO 2025a); these are referred to as'‘Unclassified’'in the ECDC antimicrobial consumption dashboard (ECDC 2025)	not classified under the AWaRe system, but essential for treating specific bacterial infections	

This study focuses on the prudent use of antibacterial drugs for the human health sector, analysing past distributions of consumption rates and comparing trends between AWaRe drug classes. Long-term ARIMA modelling is used to forecast consumption patterns in a large number of European countries and to assess whether current trends are consistent with EU targets for 2030. The study aims to assess the likelihood of meeting these targets, compare forecasts with antibacterial consumption and identify both rational and problematic prescribing practices in order to generalise regional patterns.

Previous research by our group has identified key factors influencing consumption trajectories, for example costs, guideline changes, rates and changes in bacterial resistance, side effect concerns or policy changes (Bindel and Seifert 2024a, 2024b, 2024c, 2024 d) and revealed significant correlations between antibacterial consumption and AMR (Bindel and Seifert 2024c). It has also highlighted instances of irrational prescribing practices (Bindel and Seifert 2024b, 2024c, 2024 d) and successfully predicted future consumption of antibacterials and other drug classes using ARIMA models (Bindel and Seifert 2025a, 2025b, 2025c). These results provide a basis for understanding the factors that could lead to increasing or decreasing consumption patterns and potential drivers of irrational prescribing behaviour, for example, when treatment choice is based on lowest costs rather than pathogen susceptibility (Bindel and Seifert 2024a, 2024b, 2024c, 2024 d).

While many existing studies focus on past trends (ECDC 2024a), this study provides a forward-looking approach. Prescribing behaviour is often characterised only by total consumption, which does not sufficiently distinguish between rational and problematic practices (Robertson et al. 2021). By combining qualitative assessment with quantitative analysis (Bindel and Seifert 2025b), this study provides a comprehensive view of antibacterial drug consumption. Projecting the consequences of current prescribing practices aims to inform long-term planning, supporting efforts to increase rational use and identify particularly problematic regions and drug groups.

## Methods and materials

### Setting and data

This study analyses the distribution of WHO AWaRe ('Access','Watch','Reserve') antibacterial drug groups (WHO 2023) in 30 European countries using data from the ECDC Antimicrobial Consumption Dashboard (https://qap.ecdc.europa.eu/public/extensions/AMC2_Dashboard/AMC2_Dashboard.html#eu-consumption-tab) (ESAC-net 2025), covering the years 1997–2023. An additional group'Unclassified'was introduced for drugs not categorised under the AWaRe framework (ESAC-net 2025). Consumption is expressed as proportions and defined daily doses per 1000 inhabitants per day (DID). Data is covering both community and hospital sectors for all countries except Germany, where only the community sector is regarded. There are differences in data availability and covered population (Table S1). The AWaRe framework includes antibacterials, but no antifungals, antiparasitics or antivirals.

**Fig. 1:**
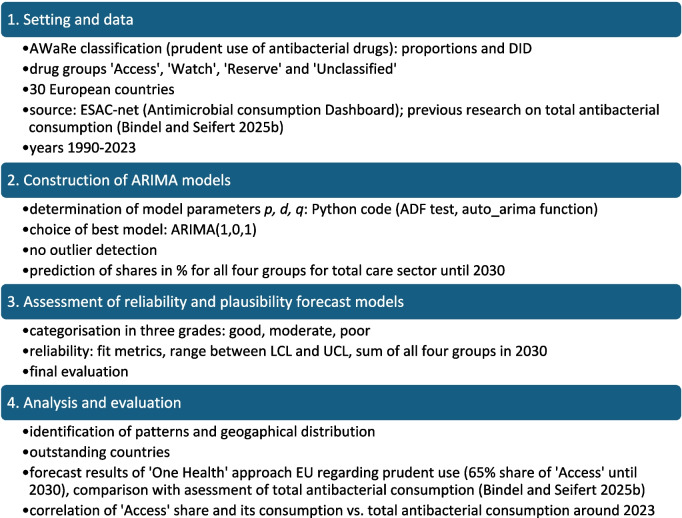
Methodical approach for time series analysis in SPSS with the ARIMA-model to predict futural development of DDD-prescriptions for antibacterial drugs

The methodological procedure is illustrated in Fig. 1.

### Prediction with ARIMA models

The autoregressive integrated moving average (ARIMA) model was chosen for its effectiveness in forecasting drug use trends (Hyndman and Athanasopoulos 2021; Bindel and Seifert 2025a). ARIMA(*p,d,q*) includes parameters to account for autoregression (*p*), differencing (*d*) for stationarity, and moving average (*q*) to model past errors (Nau 2020; Box et al. 2015; NIST/SEMATECH 2012). Model selection was based on the Bayesian Information Criterion (BIC). The Augmented Dickey-Fuller (ADF) test was used to assess stationarity (Dickey and Fuller 1979). Country-specific ARIMA models were developed alongside a global model applied to the combined dataset, with the global ARIMA(1,0,1) model emerging as the best fit.

To determine the optimal ARIMA parameters for different time series, the analysis used several Python libraries, including ‘pandas’ (McKinney 2010), ‘pmdarima’ (pypi 2023), ‘statsmodels’ (Seabold and Perktold 2010) and ‘openpyxl’ (pypi 2024). The code was executed in Colab (https://colab.research.google.com/). Forecasts were generated in SPSS for each country, automatic outlier detection was tested but found unnecessary. The projections extend to 2030, with confidence intervals indicating uncertainty. The EU's One Health target (EU 2023) was assessed with the 2030 projection, but only for human health and not for all'One Health'sectors.

### Assessment of fit metrics and reliability

The assessment of reliability is based on the model fit metrics stationary R-squared, R-squared, MAPE and MaxAPE of the ‘Access’ group. A good fit is considered as stationary R-squared above 0.65, R-squared above 0.85, MAPE below 6 and MaxAPE below 15. Moderate fit includes R-squared between 0.4 and 0.65, R-squared between 0.6 and 0.84, MAPE between 7 and 20 and MaxAPE between 16 and 40. Poor fit is indicated by R-squared less than 0.4, R-squared less than 0.6, MAPE greater than 20 and MaxAPE greater than 40.

Given the relatively limited data set, the predictive power of the model is restricted. Therefore, more emphasis should be placed on the reliability of trends rather than the precise prediction of specific values in the long term (Bindel and Seifert 2025a, 2025b). Furthermore, the forecast is based on past data and therefore predicts future trends on the assumption that past trends will continue in the future. Changing circumstances or sudden events can't be foreseen.

### Correlation analysis and comparison with total consumption volume

Pearson correlation was used to assess the relationships between the distribution of AWaRe groups and total antibacterial consumption (Bindel and Seifert 2025b; Mukaka 2012), performed in SPSS. Correlations above ± 0.8 were considered strong. Statistical significance was set at p < 0.05.

## Results

### ‘Access’ share: past development and prediction until 2030

The WHO AWaRe framework (Table 1) is a valuable tool for assessing the quantity of antibacterial prescribing for further qualitative assessment, covering around 48% of common quality indicators (Funiciello et al. 2024). The proportion of the'Access'group is an indicator of prudent antibacterial drug use (ECDC 2024a), with high proportions being favourable (EU 2023), while lower proportions signal potentially problematic prescribing behaviour. In the following, the past development and the forecast up to 2030 are presented for the'Access'proportion of each European country analysed. More detailed information can be found in Tables 2 and 3.

**Table 2:**
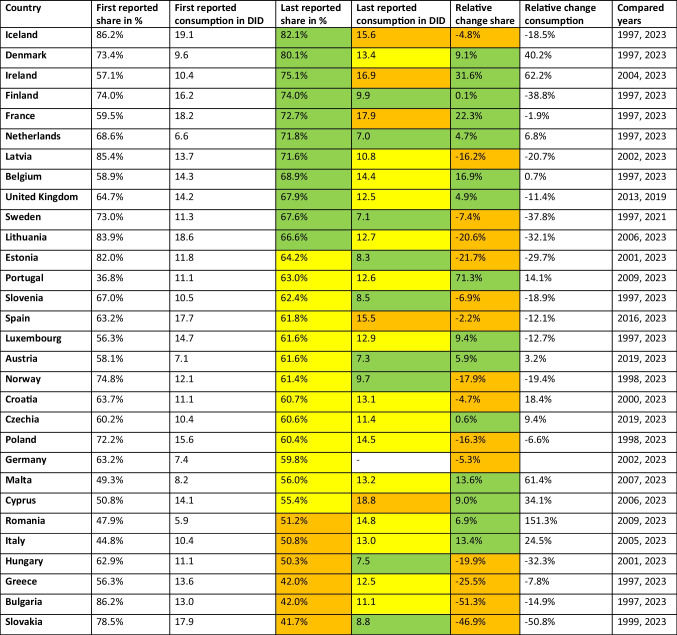
Development of the'Access'group. The first reported shares and consumption are given and compared with the most recent share and consumption data. The relative proportions of consumption and proportions are given, as well as the years compared. For recent shares, a share above 65% is considered good and coloured green, a share between 55–65% is considered moderate and coloured yellow, and a share below 55% is considered poor and coloured orange. In 2023, consumption below 10 DID is considered good and coloured green, between 10–15 DID moderate and coloured yellow, while above 15 DID is poor and coloured orange. Increasing proportions are considered good and coloured green, while decreasing proportions are problematic and coloured orange. Countries are sorted descending by their share around 2023

**Table 3:**
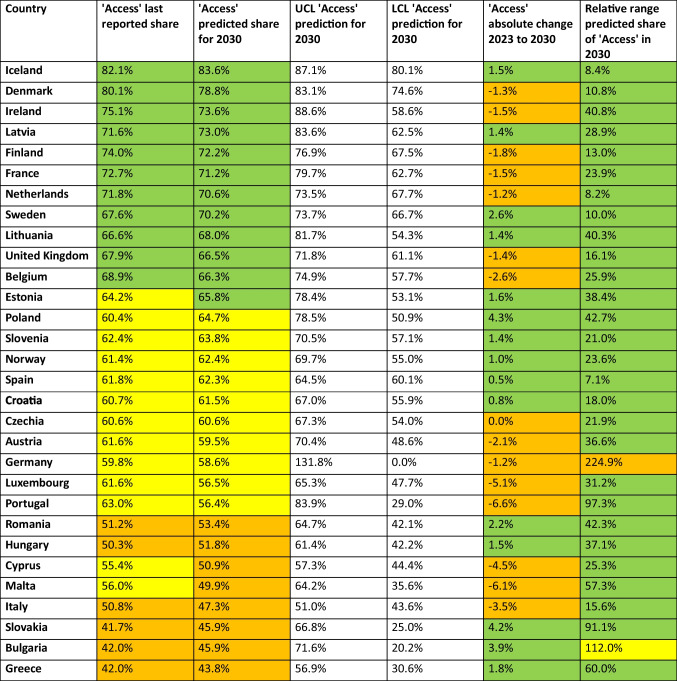
Prediction of the share of the'Access'group until 2040. The lastly reported shares around 2023 and the prediction, UCL and LCL for 2030 are given. Calculated is the absolute change of the Access group from 2023 - 2030 and the relative range of the UCL and LCL in 2030. A share above 65% is considered good and coloured green, a share between 55–65% is considered moderate and coloured yellow, and a share below 55% is considered poor and coloured orange. Consumption below 10 DID is considered good and coloured green, between 10–15 DID moderate and coloured yellow, while above 15 DID is poor and coloured orange. Increasing proportions are considered good and coloured green, while decreasing proportions are problematic and coloured orange. Countries are sorted descending by their predicted share in 2030

For the latest reported data, mostly for years around 2023, the'Access'share varies from 82.1% to 41.7% (Table 2). 11 countries exceed the 65% threshold of the EU (EU 2023), including Iceland (82.1%), Denmark (80.1%), Ireland (75.1%), Finland (74.0%), France (72.7%), the Netherlands (71.8%), Latvia (71.6%), Belgium (68.9%), the United Kingdom (67.9%), Sweden (67.6%) and Lithuania (66.6%). Shares around the average are reported for Estonia (64.2%), Portugal (63.0%), Slovenia (62.4%), Spain (61.8%), Luxembourg and Austria (61.6%), Norway (61.4%), Croatia (60.7%), Czechia (60.6%), Poland (60.4%), Germany (59.8%), Malta (56.0%) and Cyprus (55.4%). Low shares are reported of 6 countries, including Romania (51.2%), Italy (50.8%), Hungary (50.3%), Greece and Bulgaria (42.0%) and Slovakia (41.7%). Fifteen countries, including Portugal, Ireland, France, Belgium, Malta, Italy, Luxembourg, Denmark, Cyprus, Romania, Austria, the United Kingdom, the Netherlands, Czechia and Finland, show a favourable trend in recent years, i.e. an increase in the'Access'share. On the other hand, the other 15 countries, including Bulgaria, Slovakia, Greece, Estonia, Lithuania, Hungary, Norway, Poland, Latvia, Sweden, Slovenia, Germany, Iceland, Croatia and Spain, recorded a decrease.

In the 2030 forecast (Table 3), 12 countries have an'Access'share of more than 65%, such as Iceland (83.6%), Denmark (78.8%), Ireland (73.6%), Latvia (73.0%), Finland (72.2%), France (71.2%), the Netherlands (70.6%), Sweden (70.2%), Lithuania (68.0%), the United Kingdom (66.5%), Belgium (66.3%) and Estonia (65.8%). On the other hand, eight countries are expected to have a low share, including Greece (43.8%), Slovakia and Bulgaria (45.9%), Italy (47.3%), Malta (49.9%), Cyprus (50.9%), Hungary (51.8%) and Romania (53.4%). The other 10 countries fall in between, with shares ranging from 56.4% to 64.7%. A favourable upward trend is forecast for 14 countries, including Poland, Slovakia, Bulgaria, Sweden, Romania, Greece, Estonia, Iceland, Hungary, Latvia, Slovenia, Lithuania, Norway, Croatia and Spain. On the other hand, the share is expected to fall in the 15 countries Portugal, Malta, Luxembourg, Cyprus, Italy, Belgium, Austria, Finland, Ireland, France, the United Kingdom, Denmark, Germany and the Netherlands. The only country expected to remain unchanged is Czechia.

### ‘Watch’, ‘Reserve’ and ‘Unclassified’ shares: past development and prediction until 2030

Antibacterial substances that should be used restrictively include the'Watch'and'Reserve'groups of the AWaRe framework (WHO 2023), with a further reduction in their proportion and consumption favoured to reduce the rise of AMR (Lee et al. 2013; ECDC 2024a). The'Watch'group includes substances reserved for severe infections and therefore their use should remain at low levels (ECDC 2024a). The'Reserve'group includes antibacterial treatment of last resort and should be used minimally (WHO 2023). The'Unclassified'group includes antibacterial substances that have not yet been categorised. Low proportions can be considered unremarkable, while higher proportions indicate potential gaps in the current AWaRe classification. In the following, the historical development and the forecast up to 2030 are presented for the shares of each European country analysed. More detailed information can be found in Tables 4, 5, 6, and 7.

**Table 4:**
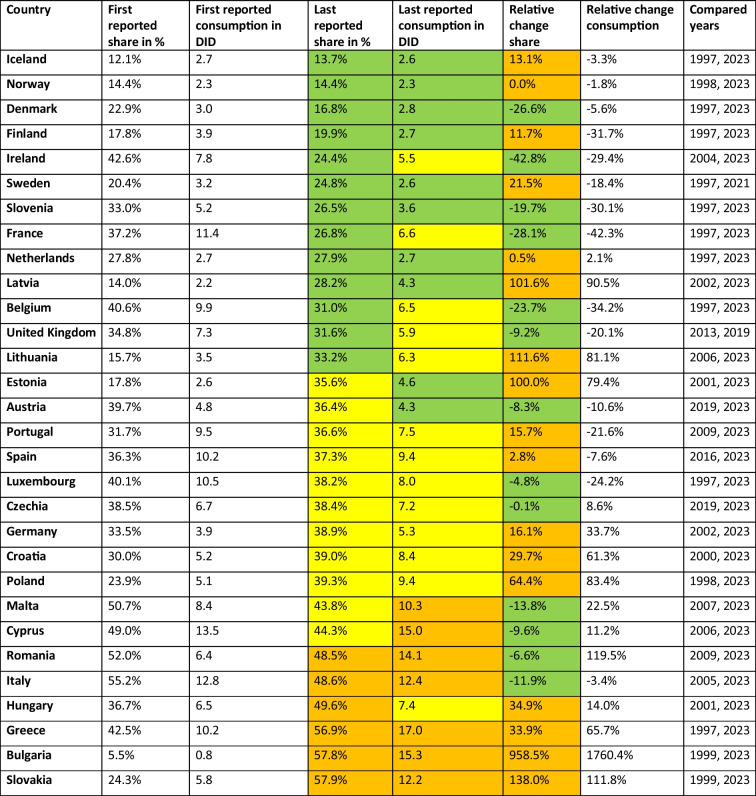
Development of the'Watch'group. The first reported shares and consumption are given and compared with the most recent share and consumption data. The relative proportions of consumption and proportions are given, as well as the years compared. For recent shares, a share under 35% is considered good and coloured green, a share between 35–45% is considered moderate and coloured yellow, and a share over 45% is considered poor and coloured orange. In 2023, consumption below 5 DID is considered good and coloured green, between 5–10 DID moderate and coloured yellow, while above 10 DID is poor and coloured orange. Decreasing proportions are considered good and coloured green, while increasing proportions are problematic and coloured orange. Countries are sorted ascending by their share around 2023

**Table 5:**
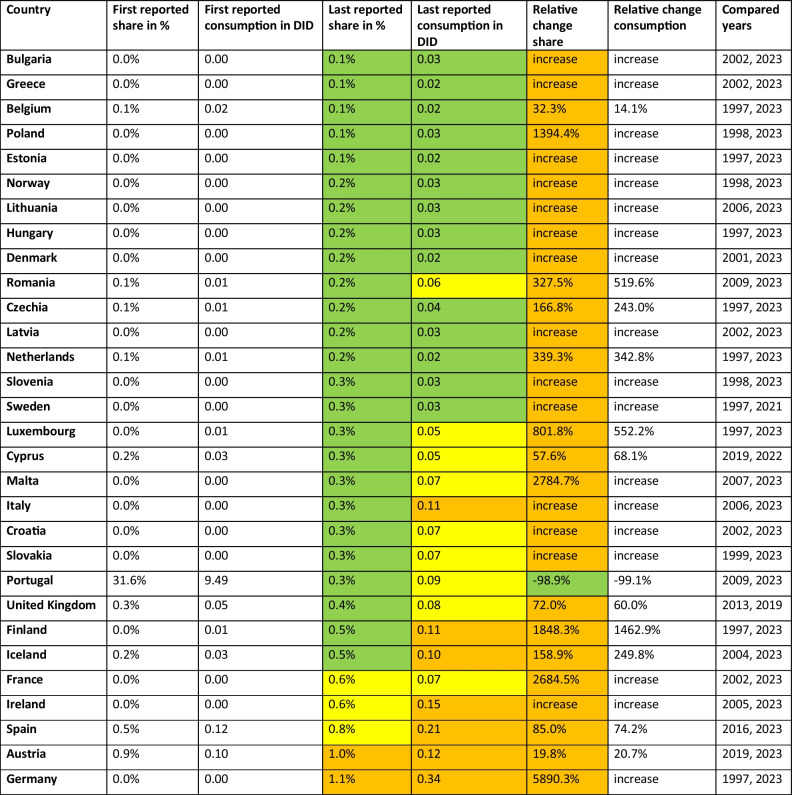
Development of the'Reserve'group. The first reported shares and consumption are given and compared with the most recent share and consumption data. The relative proportions of consumption and proportions are given, as well as the years compared. For recent shares, a share under 0.5% is considered good and coloured green, a share between 0.5–1.0% is considered moderate and coloured yellow, and a share over 1.0% is considered poor and coloured orange. In 2023, consumption below 0.05 DID is considered good and coloured green, between 0.05–0.1 DID moderate and coloured yellow, while above 0.1 DID is poor and coloured orange. Decreasing proportions are considered good and coloured green, while increasing proportions are problematic and coloured orange. Countries are sorted ascending by their share around 2023

**Table 6:**
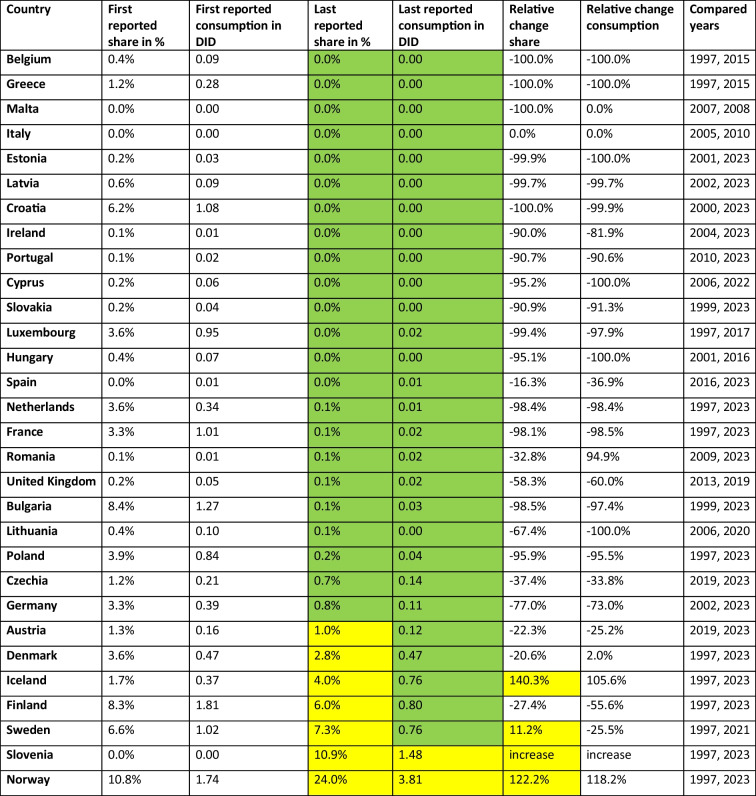
Development of the'Unclassified'group. The first reported shares and consumption are given and compared with the most recent share and consumption data. The relative proportions of consumption and proportions are given, as well as the years compared. For recent shares, a share under 1.0% is considered good and coloured green, while a share over 1.0% is unusual high and yellow-coloured. In 2023, consumption below 0.1 DID is considered unremarkable and coloured green, while above 1.0 DID is being remarkable and coloured yellow. Increasing proportions depict unusual behaviour and are therefore yellow-coloured. Countries are sorted ascending by their share around 2023

**Table 7:**
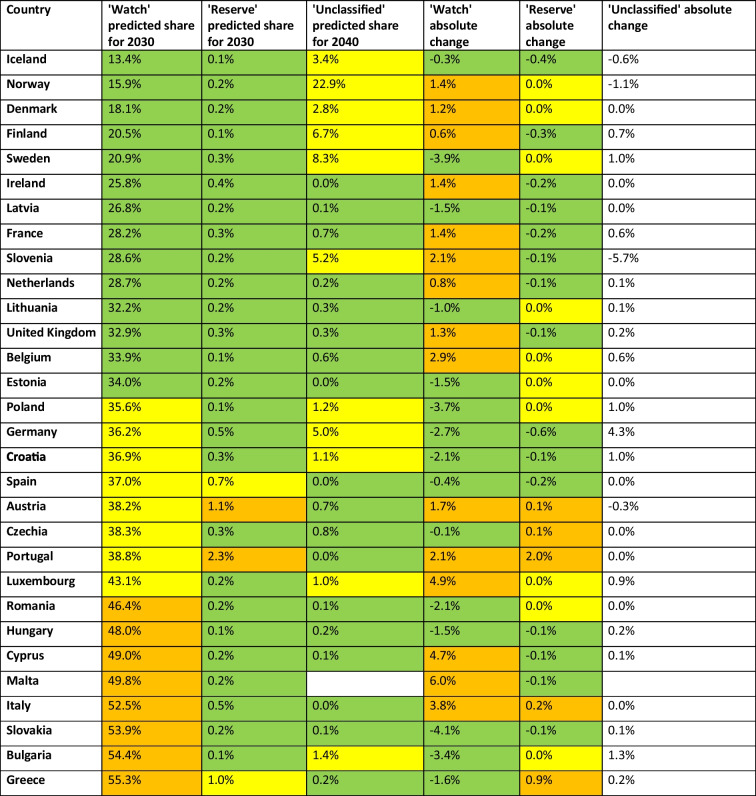
Prediction of the share for the'Watch’, ‘Reserve’ and ‘Unclassified’ group until 2030. The predicted shares for 2030 and the absolute change are given for each group. Values are green-coloured if the share is considered good (until 35% in ‘Watch’, until 0.5% in ‘Reserve’), yellow when it is considered moderate (35–45% in ‘Watch’, 0.5–1.0% in ‘Reserve’) and orange when it is considered poor (exceeding 45% in ‘Watch’, exceeding 1.0% in ‘Reserve’). Within the ‘Unclassified’ group, an absolute change exceeding + 1.0% is considered remarkable and therefore yellow-coloured. Decreasing absolute changes are considered fortunate and green-coloured, while increases are considered problematic and orange-coloured for the ‘Watch’ and Reserve’ group. Countries are sorted ascending by their predicted share of the ‘Watch’ group in 2030

Recently reported'Watch'proportions (Table 4) are considered low, i.e. below 35%, for 13 countries, including Iceland, Norway, Denmark, Finland, Ireland, Sweden, Slovenia, France, the Netherlands, Latvia, Belgium, the United Kingdom and Lithuania. Around the average are Estonia, Austria, Portugal, Spain, Luxembourg, Czechia, Germany, Croatia, Poland, Malta and Cyprus. Notably high proportions are reported for 6 countries, including Romania, Italy, Hungary, Greece, Bulgaria and Slovakia. In past years, the share decreased in 13 countries, including Ireland, France, Denmark, Belgium, Slovenia, Malta, Italy, Cyprus, the United Kingdom, Austria, Romania, Luxembourg and Czechia, while it increased in the remaining 17 countries. The forecast for 2030 (Table 7) projects a low share (below 35%) for the 14 countries Iceland, Norway, Denmark, Finland, Sweden, Ireland, Latvia, France, Slovenia, the Netherlands, Lithuania, the United Kingdom, Belgium and Estonia. Countries with a moderately higher share includes the 8 countries Poland, Germany, Croatia, Spain, Austria, Czechia, Portugal and Luxembourg, which range between 35.6% and 43.1%. A high proportion (over 45%) is forecast for the eight countries Romania, Hungary, Cyprus, Malta, Italy, Slovakia, Bulgaria and Greece. In terms of expected changes, decreases are forecast for the 15 countries Slovakia, Sweden, Poland, Bulgaria, Germany, Romania, Croatia, Greece, Hungary, Estonia, Latvia, Lithuania, Spain, Iceland and Czechia, while increases are forecast for the 15 other countries.

Regarding the ‘Reserve’ share around 2023 (Table 5), for 25 countries it is very low (under 0.5%). A comparably high proportion is reported for the five countries France and Ireland (0.6%), Spain (0.8%), Austria (1.0%) and Germany (1.1%). In past years, the share increased in nearly all countries, except Portugal who reported a decreasing share. In 2030 (Table 7), predictions include a low share under 0.5% for 26 countries, while four countries are forecast with a comparably high proportion, including Spain (0.7%), Greece (1.0%), Austria (1.1%) and Portugal (2.3%). 15 countries will have decreased in their share, including Germany, Iceland, Finland, France, Ireland, Spain, Slovakia, Malta, the United Kingdom, Hungary, Cyprus, Slovenia, Croatia, the Netherlands and Latvia. In contrast, five countries are expected to increase, including Czechia, Austria, Italy, Greece and Portugal. For 10 countries, no change is forecast, including Romania, Luxembourg, Poland, Lithuania, Norway, Bulgaria, Sweden, Belgium, Estonia and Denmark.

The proportion of ‘Unclassified’ substances are very low (under 1%) for 23 countries in recent years (Table 6). Comparably high shares are reported for the five countries France and Ireland (0.6%), Spain (0.8%), Austria (1.0%) and Germany (1.1%). In past years, the share increased in almost all countries, with the exception of Portugal that reported a decrease. By 2030 (Table 7), 26 countries are forecast to have a low share of less than 0.5%, while four countries are forecast to have a relatively high share, including Spain (0.7%), Greece (1.0%), Austria (1.1%) and Portugal (2.3%). Fifteen countries will see their share fall, including Germany, Iceland, Finland, France, Ireland, Spain, Slovakia, Malta, the United Kingdom, Hungary, Cyprus, Slovenia, Croatia, the Netherlands and Latvia. In contrast, five countries are expected to increase, including Czechia, Austria, Italy, Greece and Portugal. The other ten countries are expected to remain unchanged.

## Discussion

### Analysis of AWaRe distribution in past years and future projections

By examining historical and recent distributions of the AWaRe groups, patterns in prudent and problematic use can be identified. The ARIMA(1,0,1) model was used to predict the future distribution until 2030, assessing whether current trends will continue in the future.

**Fig. 2:**
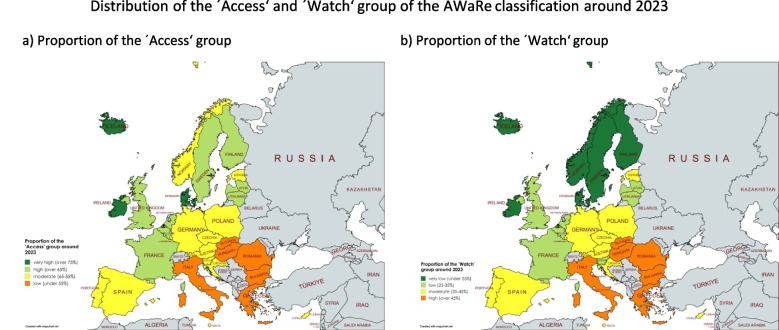
Last reported share of the ‘Access’ and ‘Watch’ group. Most countries reported data for 2023, the exceptions being Sweden with the last report in 2021 and the United Kingdom in 2019. Countries considered to have a prudent use of antibacterial drugs are green-coloured (indicated by a high share of the ‘Access’ and a low share of the ‘Watch’ group), countries with a moderate behaviour are yellow-coloured and countries with a poor behaviour are orange-coloured (indicated by a low share of the ‘Access’ group and a high share of the ‘Watch’ group). Countries where no data was available are grey-coloured. The map was created using mapchart.net

**Fig. 3:**
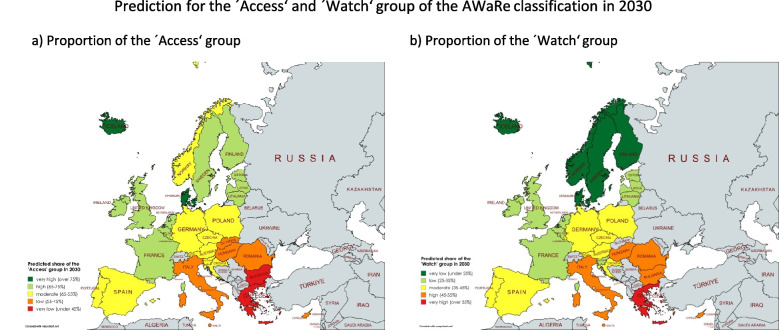
Prediction for the share of the ‘Access’ and ‘Watch’ group in 2030. Countries considered to have a prudent use of antibacterial drugs are green-coloured (indicated by a high share of the ‘Access’ and a low share of the ‘Watch’ group), countries with a moderate behaviour are yellow-coloured and countries with a poor behaviour are orange-coloured (indicated by a low share of the ‘Access’ group and a high share of the ‘Watch’ group). Countries where no data was available are grey-coloured. The map was created using mapchart.net

Patterns of antibacterial drug use are consistent across many countries (Tables 1, 2, 3, 4, 5, 6 and 7, Fig. 2–3). Recently, prudent prescribing, with a high share of'Access'(exceeding 65%) and low'Watch'(under 35%), is observed in Iceland, Denmark, Ireland, Finland, France, the Netherlands, Latvia, Belgium, the United Kingdom, Sweden and Lithuania. Moderate prescribing, meaning that the ‘Access’ share is slightly under 65% and the ‘Watch’ share is slightly exceeding 35%, occurs in Estonia, Portugal, Spain, Luxembourg, Austria, Croatia, Czechia, Poland, Germany, Malta and Cyprus. Problematic prescribing, indicated by exceptionally low'Access'and high'Watch'proportions, is seen in Romania, Italy, Hungary, Greece, Bulgaria and Slovakia. Norway and Slovenia, while differing in their'Access'shares, report exceptionally high'Unclassified'proportions (10.9% and 24.0%), complicating assessments. The'Reserve'group remains low in most countries, though Austria and Germany exceed 1.0%, despite moderate classifications in'Access'and'Watch'. The outstanding proportion of ‘Reserve’ for Germany is especially alarming (Table 5), since data only covers outpatient prescription, while most ‘Reserve’ antibacterials are used for the treatment of severe infections (WHO 2023) in the hospital setting.

Trends indicate an inverse relationship between'Access'and'Watch'shares (Tables 2 and 4). In past years, improvements, with rising'Access'and declining'Watch', are reported in Ireland, France, Denmark, Malta, Italy, Cyprus, the United Kingdom, Austria, Romania, Luxembourg and Czechia. Conversely, a concerning decline in ‘Access’ and rise in ‘Watch’ is observed in Bulgaria, Slovakia, Lithuania, Latvia, Estonia, Poland, Hungary, Greece, Croatia, Sweden, Germany, Iceland, Spain and Norway. An uncertain trend, with ‘Access’ and ‘Watch’ both increasing or decreasing, is recognized for Belgium, Slovenia, the Netherlands, Finland and Portugal. Alarmingly, almost all countries see an increase in'Reserve'antibiotics (Tables 5 and 7), confirming reports of rising last-resort drug use (Benko et al. 2022; ECDC 2024a).

Problematic prescribing is assumed for Greece, Bulgaria and Slovakia aligns because of high antibacterial consumption and bacterial resistance rates (ECDC 2024a; Spernovasilis and Tsioutis 2024). Factors such as cultural prescribing norms and regulatory gaps contribute to irrational use (Bindel and Seifert 2024c, 2025a, b, c). In contrast, Iceland, Denmark and Norway exemplify effective stewardship, backed by restrictive policies and national action plans (Ministry of Health Iceland 2024; Ministry of Health Denmark 2017; Gutema et al. 2021; Hobaek and Lie 2019). Many European countries have implemented strategies to improve antibacterial use (ECDC 2023). For example, countries that have strengthened their efforts to reduce antibacterial consumption have been able to successfully influence consumption trends (Oberjé et al. 2017), exhibiting better prescribing behaviour than other neighbouring countries. Examples are Scotland (MacBride-Stewart et al. 2021), Slovenia (Fürst et al. 2015) and Srpska (Bojanic et al. 2018). In contrast, countries considered to have underdeveloped strategies have higher consumption and more irrational prescribing behaviour than neighbours (Wojkowska-Mach et al. 2018).

Regarding future trends, there are both concerning as well as positive trends predicted (Table 7). Improvements, indicated by increasing ‘Access’ and decreasing ‘Watch’ shares, are projected for Iceland, Latvia, Sweden, Lithuania, Estonia, Slovenia, Spain, Croatia, Poland, Romania, Hungary, Greece, Bulgaria and Slovakia. In contrast, worsening trends are predicted for Italy, Cyprus, Malta, Austria, Luxembourg, Portugal, the United Kingdom, Belgium, the Netherlands, France, Finland, Ireland and Denmark. For some countries, the trends are inconclusive, because ‘Access’ and ‘Watch’ are changing similarly, including Norway, Czechia and Germany. Overall, the continuing increase in'Reserve'shares raises significant concerns for future resistance management.

Further details on specific country patterns and outstanding countries can be found in the Supplement.

### Reliability of forecasts

The goodness of fit for the ARIMA(1,0,1) is based on the metrics stationary R-squared, R-squared, MAPE and MaxAPE of the'Access'group (Table 8 and S5-S8). Good model performance is reported for Belgium, Croatia, Denmark, Estonia, France, Hungary, Ireland, Malta, the Netherlands, Norway and Poland. Moderate fit metrics are observed for Bulgaria, Finland, Greece, Iceland, Latvia, Lithuania, Luxembourg, Slovakia, Sweden and the United Kingdom. Poor fit metrics are suggested for Austria, Cyprus, Czechia, Germany, Italy, Portugal, Romania, Slovenia and Spain. The precision of the model is assessed by examining the sum of the predictions across drug groups and the relative range between LCL and UCL in 2030. A very accurate sum (100% ± 1%) is predicted for 25 countries, with small deviations (100% ± 2%) in Norway and Poland and larger deviations (100% ± 3%) in Portugal and Slovenia. The majority of countries have a narrow confidence range (below 100%), except for Bulgaria (112.0%) and Germany (224.9%).

**Table 8:**
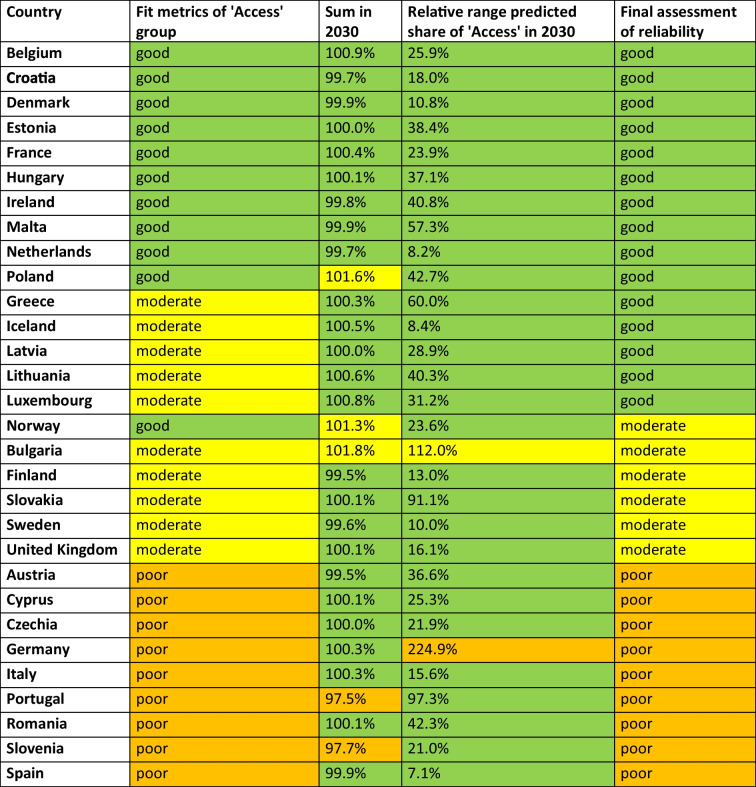
Reliability of the forecasts with a focus on the ‘Access’ group. Criteria include the assessment of fit metrics, the sum of all drug groups in 2030 and the relative range of the confidential interval for the ‘Access’ group in 2030. A model is considered to have a good fit if at least three of the four values (R-squared, R-squared, MAPE and MaxAPE) are good, moderate if at least three of the four indicators are good or moderate and poor if there are two or more values classified as poor. For the sum of all AWaRe categories in 2030, values within ± 1% of 100% are considered good, a deviation of ± 1% to 2% is considered moderate and a deviation of ± 2% to 3% is considered poor. In terms of the relative range of the confidential interval (sum of UCL – LCL), values below 100% are considered good, between 100–200% as moderate and above 200% as poor. Given is a final assessment. A country is rated as having good reliability (green-coloured) if all aspects are rated as good, and as having moderate reliability (yellow-coloured) if at least one aspect is rated as moderate. Countries with a poor rating (orange-coloured) are those with one aspect rated poor. Countries are sorted descending by their final assessment and consideration of fit metrics

The confidence interval serves as an indicator of model uncertainty, while the predicted sum helps to assess whether drug groups are moving in parallel or showing signs of over- or under-estimation. Longer data coverage is generally associated with better model fit, while poor data quality leads to weaker performance. The stability of time series trends also affects reliability, as abrupt changes or unstable patterns introduce uncertainty into future projections (Bindel and Seifert 2025a). Narrow confidence intervals suggest less variation in possible outcomes, whereas wider ranges are often associated with moderate or poor fit metrics (Table 8). Furthermore, while fit metrics help assess model adequacy, even an imprecisely fitted model can produce seemingly accurate but misleading predictions (Bindel and Seifert 2025a). Notably, despite independent predictions for each drug classification, most countries show highly consistent predictions.

A final assessment of model performance classifies 15 countries as having accurate and reliable forecasts, including Belgium, Croatia, Denmark, Estonia, France, Greece, Hungary, Iceland, Ireland, Latvia, Lithuania, Luxembourg, Malta, the Netherlands and Poland. Moderate reliability is attributed to Bulgaria, Finland, Norway, Slovakia, Sweden and the United Kingdom, where trends are well captured but some aspects lack precision. Low reliability is observed in Austria, Cyprus, Czechia, Germany, Italy, Portugal, Romania, Slovenia and Spain, where data quality might affect the adaptability of the model. A poor classification does not imply incorrect forecasts, but rather reflects uncertainty, as seen in deviating sums or wide confidence intervals. Nevertheless, even these less reliable forecasts provide insight into future trends.

Beside model performance, projections are limited by the reliance on historical data to project long-term developments, as unforeseen events can change trends (Bindel and Seifert 2025a). The projections assume persistent trends, stable external factors and unchanged prescribing behaviour. However, future policy changes, the evolution of bacterial resistance (European Antimicrobial Resistance Collaborators 2022), shifts in demand due to infectious disease outbreaks (Nandi et al. 2023; CDC 2025), healthcare accessibility, sanitation and drug availability in conflict zones (Geddes 2024), or breakthroughs in antibacterial and non-antibacterial treatments (Dance 2024; Nature 2024) remain unpredictable.

### Geographical distribution of drug prescribing behaviour

The geographical distribution of the AWaRe-classified shares reveals different patterns of antibacterial drug use across Europe, which can be broadly divided into five regions: Central, North, South, West and East. An evaluation of these trends highlights both strong contrasts and notable similarities in prescribing behaviour across these regions (Fig. 2 and 3, Table 9).

**Table 9:**
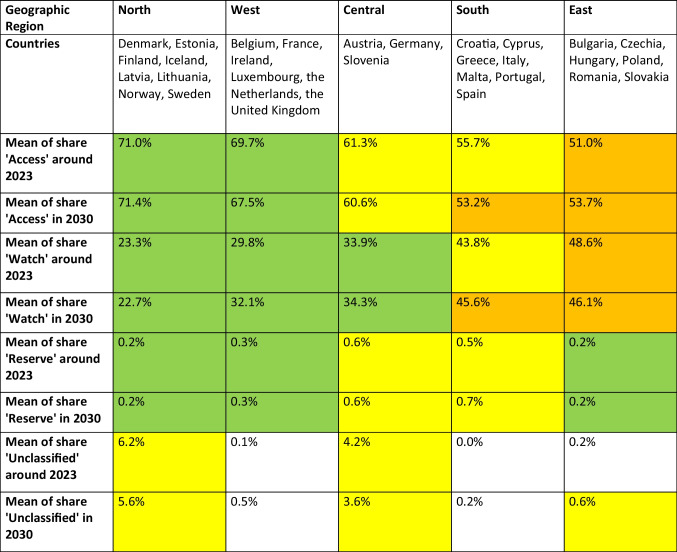
Geographic distribution of current and predicted shares ofl AWaRe classification groups. Values are green-coloured if the share is considered good (until 35% in ‘Watch’, until 0.5% in ‘Reserve’), yellow when it is considered moderate (35–45% in ‘Watch’, 0.5–1.0% in ‘Reserve’) and orange when it is considered poor (exceeding 45% in ‘Watch’, exceeding 1.0% in ‘Reserve’). Within the ‘Unclassified’ group, an absolute change exceeding + 1.0% is considered remarkable and therefore yellow-coloured. Regions are sorted descending by their last reported share of the ‘Access’ group

The'Access'group comprises a higher share of total antibacterial consumption in Northern (71.0%) and Western (69.7%) Europe compared to Southern (55.7%) and Eastern (51.0%) Europe around 2023. In 2030, the forecast models predict an average share of 71.4% for North, 67.5% for West, 60.6% for Central, 53.2% for South and 53.7% for East Europe. This means that Northern and Eastern regions can improve their overall prescription quality, while Western, Central and Southern Europe will worsen.

The'Watch'group is recently used restrictively in Northern (23.3%), Western (29.8%) and Central (33.9%) Europe, but is more frequently used in Southern Europe (43.8%) and worryingly high in Eastern Europe (48.6%). In 2030, predicted shares reach 22.7% for North, 32.1% for West, 34.3% for Central, 45.6% for South and 46.1% for East Europe. This indicates that fortunate declining trends are apparent for North and East Europe, while problematic increasing trends are predicted for West, Central and South Europe, similar to the ‘Access’ group.

The'Reserve'group, which consistently has the lowest share of AWaRe categories, is found at low levels in Northern (0.2%), Western (0.3%) and Eastern (0.2%) Europe around 2023. In contrast, Central (0.6%) and Southern (0.5%) Europe show moderate levels. In 2030, shares will not change for North, West, Central and East Europe. In contrast, South Europe will increase to 0.7%, exacerbating an already worrying situation and continuation of trends from recent years (ECDC 2024a).

The'Unclassified'group poses unique challenges because it includes all antibacterial drugs that have not yet been classified in the AWaRe framework. This limitation makes it difficult to assess whether their use is rational or problematic and highlights gaps in the WHO classification system that require further refinement. Western (0.1%), Eastern (0.2%) and Southern (0.0%) Europe report low and unremarkable proportions of'Unclassified'substances. However, Central (4.2%) and Northern (6.2%) Europe have recently recorded significantly high proportions. Proportions might increase in West (0.5% in 2030), South (0.2%) and East (0.6%) Europe, while decreasing in North (5.6%) and Central (3.6%) Europe. Nevertheless, elevated levels will persist in Central and Northern Europe, raising concerns about whether the current classification adequately covers all antibacterial drug use.

In summary, the geographical distribution of AWaR re-classified proportions shows a north–south gradient in antibacterial drug use across Europe. Further characterisation of each region can be found in the Supplement. Northern and Western Europe show prudent use, Central Europe occupies a moderate intermediate position and Eastern and Southern Europe remains a major concern (Klein et al. 2021). Nevertheless, there are differences between countries in the same region. Projections indicate overall improvements in the North and East Europe, while worsening trends are assumed for South, West and Central Europe. Regional shifts and trends in antibacterial drug use and prescribing behaviour are confirmed by several publications (ECDC 2024a; Benkő et al. 2022; Tang et al. 2023; Adekoya et al. 2021). Furthermore, these geographical shifts and divergent trends between European countries are also observed for other classes of medicines, such as thyroid hormones (Bindel and Seifert 2025c) and for rational prescribing behaviour of medicines in general (Rotar et al. 2020; Tian et al. 2023). In sum, there is no overall trend towards improvement, and the poor progress of recent years is expected to continue (ECDC 2024a).

### Progress towards EU target of 65% ‘Access’ share and comparison with target of − 20% antibacterial consumption

The growing problem of bacterial resistance to antibacterial drugs is being addressed by international health organisations through action plans to support rational prescribing. Many organisations have set targets for their members to achieve, often referring to the'One Health'approach. Targets include a reduction in total antibacterial consumption, a reduction in bacterial resistance and the AWaRe framework, having to be achieved until 2030 (EU 2023; ECDC 2024a; Bindel and Seifert 2025b). Notably, organisations have different targets. WHO has the lowest thresholds and wants its members to reach at least 60% of the'Access'group (Zanichelli et al. 2023; WHO 2025). The EU has set its target at 65% (EU 2023) and the UN at 70% (UN 2024). Differences in the targets may be explained by differences in the Member States, i.e. WHO wants to set a minimum standard in view of the global situation, the EU wants to adapt its targets to the European situation and the UN wants to put pressure on its members to respond to increasing AMR. As the countries analysed so far are European, the 65% access target of the EU (EU 2023) is assessed. However, using the Table 10 provided, countries can be assessed for other thresholds.

**Table 10:**
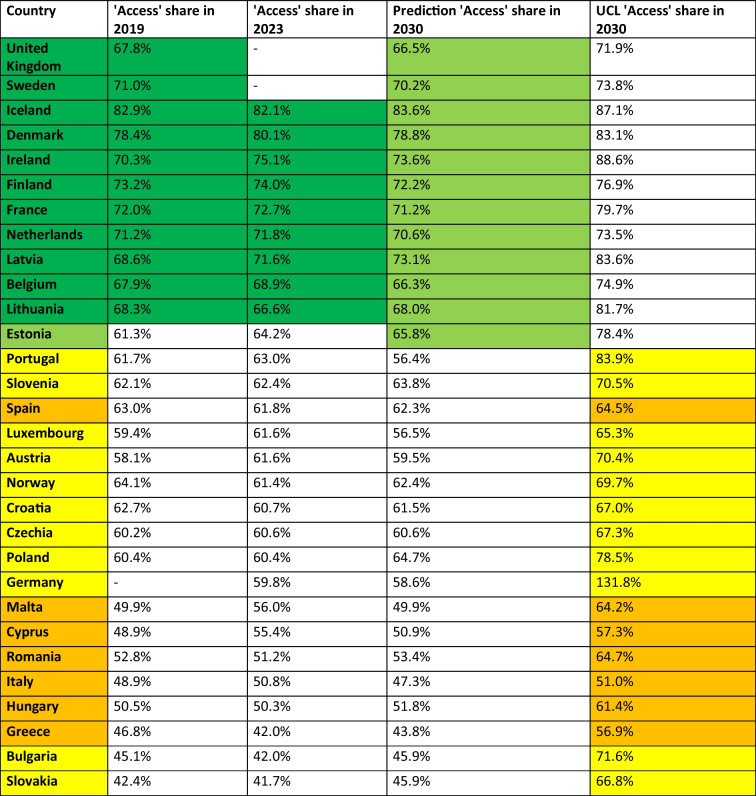
Projections for the EU'One Health'target of at least 65% share of'Access'by 2030. Shown is the share in 2019 (baseline), 2023 (last reported consumption) and the projection and UCL for 2030. Dark green highlights countries that already meet the target and are projected to do so in 2030. Light green highlights countries that are projected to meet the target by 2030 but have not recently done so. Yellow indicates countries that are projected to miss the target, but are still projected to reach it within the confidence interval. Orange highlights countries that are projected to miss the target. Countries are sorted descending by their share in 2023

A number of countries have already reached the target, when looking at the latest reported proportion of the ‘Access’ group. This includes Iceland, Denmark, Finland, France, the Netherlands, Sweden, Ireland, Latvia, Lithuania, Belgium and the United Kingdom. All these countries are expected to meet the target in 2030 as well.

Worryingly, there is only one country, Estonia, which is projected to reach the target in 2030 while not fulfilling it in 2019 or 2023. Estonia had a share of 61.3% in 2019, 64.2% in 2023 and is projected to reach 65.8% in 2030. All other countries that don't reach the target in 2019 or 2023 are projected to fail to reach the required share in 2030. These include Norway, Spain, Croatia, Slovenia, Portugal, Poland, Czechia, Luxembourg, Austria, Romania, Hungary, Malta, Italy, Cyprus, Greece, Bulgaria, Slovakia and Germany.

Considering the'best case'scenario, represented by the LCL of the ARIMA(1,0,1) model, some other countries might have the potential to reach the target of 65% of the ‘Access’ group by 2030. These include Luxembourg, Slovakia, Croatia, Czechia, Norway, Austria, Slovenia, Bulgaria, Poland, Portugal and Germany. On the other hand, for several countries the model excludes any possibility of reaching the required proportion. These include Italy, Greece, Cyprus, Hungary, Malta, Spain and Romania.

Total antibacterial consumption has to be considered in the assessment of rational prescribing behaviour, beside the share of the ‘Access’ group. A comparison of current and projected results of ‘Access’ shares and total consumption (Bindel and Seifert 2025b) indicates that countries progressing toward the 65% threshold of the EU (EU 2023) often exhibit a simultaneous reduction in total consumption. Worryingly, only Sweden is projected to reach both of the two EU targets of the 65% ‘Access’ share and 20% reduction of antibacterial consumption until 2030 (Table 11).

**Table 11:**
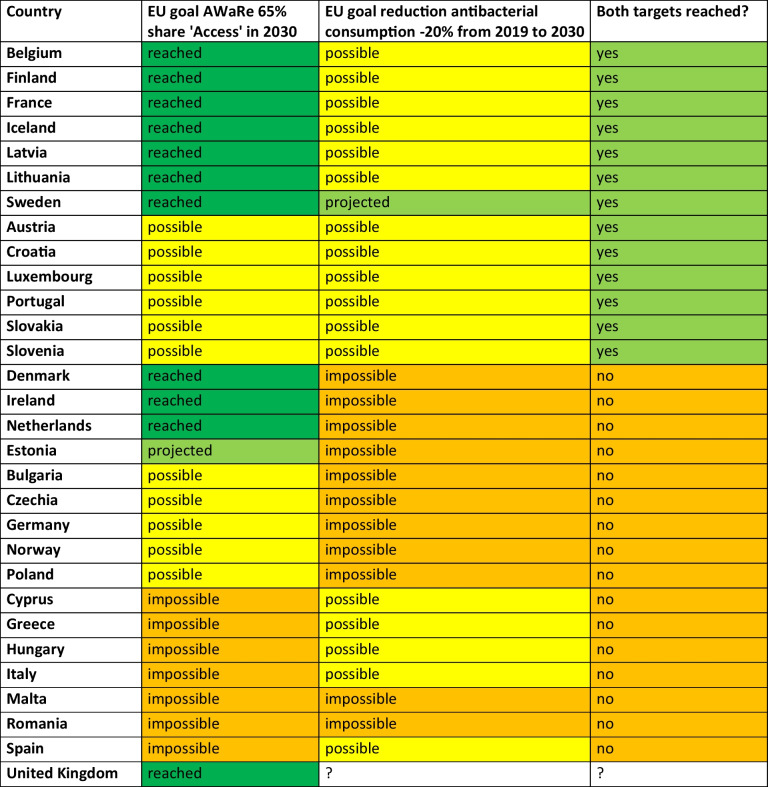
Comparison of progress towards prudent antibacterial use by EU ‘One Health’ targets (EU 2023) for the human sector. Progress is compared by predicting the share of the'Access'group and total antibacterial consumption (Bindel and Seifert 2025a, b, c). Dark green are values that confirm that the target has already been achieved. Light green are values that are projected to reach the target by 2030. Yellow are characteristics that do not reach the target within the projection, but remain possible within their confidence interval. Orange is for those for which the forecast excludes success. In the final assessment, countries where it is possible to meet both targets are coloured yellow, while countries where it is impossible to meet at least one of the two targets compared are coloured orange. Countries are ranked according to the achievement of both targets and success in the AWaRe target

**Fig. 4:**
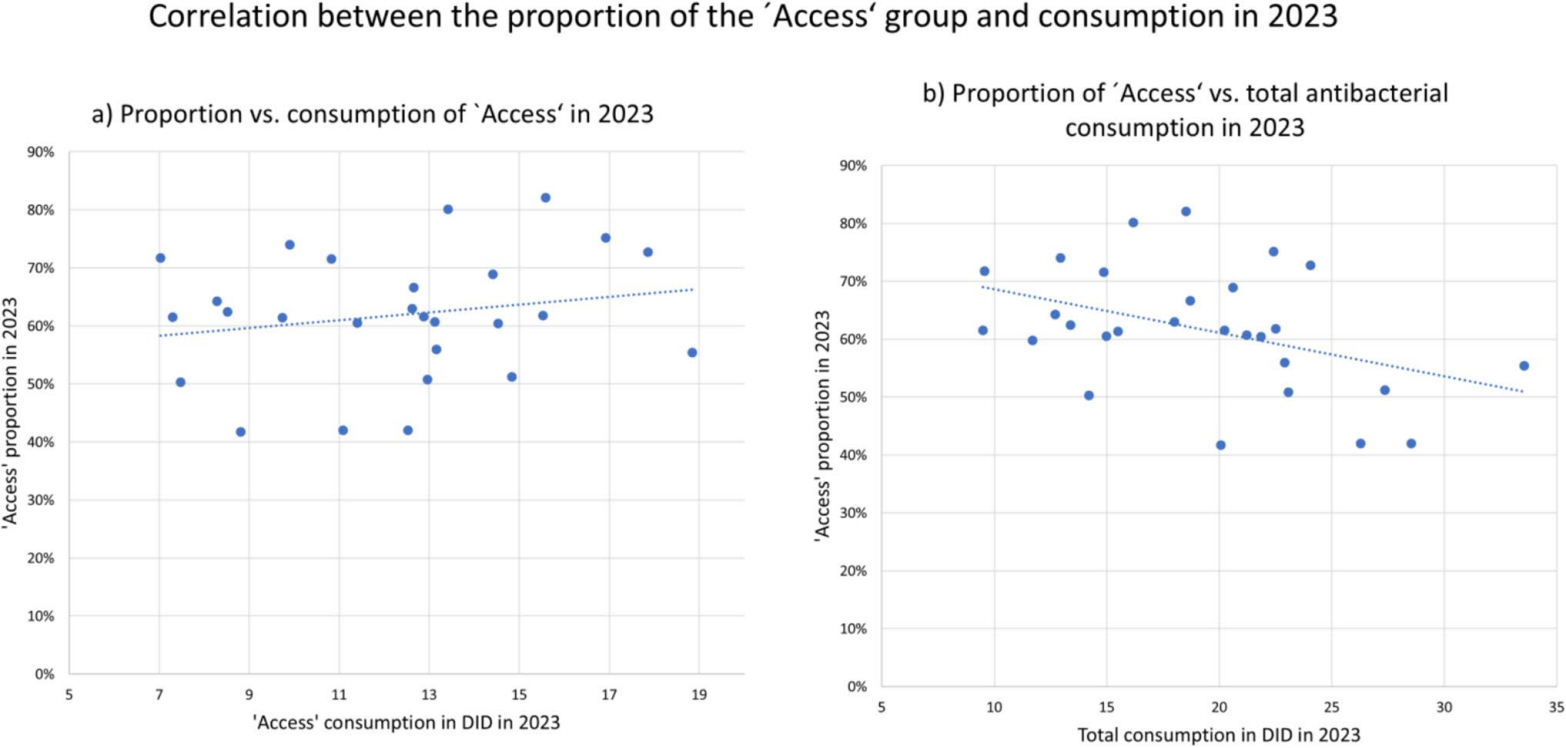
Correlation between the'Access'group and **a**) its share and **b**) total antibacterial consumption in 2023. While **a**) is not considered significant, **b**) is considered significantly negative

An analysis of the correlation between ‘Access’ share and total consumption further underscores the relationship (Table 12 and Fig. 4). A significant negative correlation was found (r = − 0.415, p = 0.028), suggesting that higher ‘Access’ shares tend to be associated with lower overall consumption. However, there is no significant correlation between the consumption and the proportion of the ‘Access’ share (r = 0.198; p = 0.322). Detailed correlation analyses are provided in the Supplement.

**Table 12:**
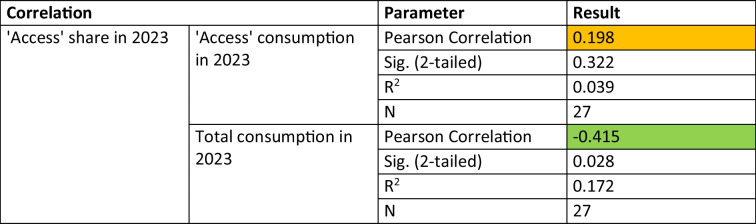
Correlations between the share of the'Access'group with its own consumption and the total consumption of antibacterial drugs (Bindel and Seifert 2025b). Orange indicates non-significant correlations and light green indicates significant correlations at the 0.05 level

In summary, the projected progress for problematic countries is poor, minimising the likelihood of achieving the EU One Health target on prudent use of antibacterial drugs (ECDC 2024c). This unfortunate trend has already been observed for the period 2019–2023 (ECDC 2024a) with particularly problematic South-Eastern regions in terms of current antibacterial drug use and low progress towards more prudent use. Referring to the 65% ‘Access’ target of the EU (EU 2023), 11 countries already achieved the target in the past, with one additional country is projected to achieve the required proportion by 2030. For 8 countries, the model offers the possibility of success within the confidential interval, while 7 countries are projected not to reach the target by 2030. In contrast, when regarding the achievement of the two targets of AWaRe distribution and reductions in total consumption, only Sweden is projected to succeed.

## Limitations

This study relies on data from the ECDC Antimicrobial Consumption Dashboard (ESAC-net 2025), which aggregates national reports. Variations in methodologies, data collection and prescribing areas across countries were not standardised, potentially introducing inconsistencies. Periodic updates of the WHO AWaRe classification (WHO 2023) may lead to discontinuities. Despite testing models with and without outlier detection, no improvement in reliability was found and the risk of overfitting led to the decision to exclude outlier correction. Data availability was a challenge, with some countries reporting limited data points (Table S1). These countries were considered to have poor data quality, but were still included for a comprehensive regional coverage. A relatively short observation period required a simpler modelling approach, preventing the inclusion of dependent variables, such as other AWaRe classification groups. The choice of ARIMA(1,0,1) was based on plausibility and model performance. Alternative statistical approaches, modifications to the model or adjustments to the fit metrics could lead to different results.

The study focused on predicting the proportions of the AWaRe groups rather than the overall evolution of DID to emphasise qualitative prescribing trends. A separate publication (Bindel and Seifert 2025b) covers total consumption projections. The AWaRe classification has limitations, expressed by the'Unclassified'category, which lacks transparency about the antibacterial drugs included. This is particularly problematic for countries such as Norway, where high proportions of unclassified substances make it difficult to evaluate qualitative drug use.

Although ARIMA models are widely used for time series forecasting (Hyndman and Athanasopoulos 2021; Bindel and Seifert 2025a), they assume linear relationships and may not fully capture complex patterns. The chosen ARIMA(1,0,1) does not take into account external factors that may influence antibacterial consumption, it is assumed that past trends will continue in the future. Long-term projections up to 2030 carry uncertainties, serving as indicative trends rather than precise forecasts.

## Conclusions and further perspectives

Historical and predicted distribution indicates a shift in antibacterial prescribing practice across Europe. While northern Europe generally maintains favourable patterns, concerning trends persist in south-eastern Europe (Tables 1, 2, 3, 4, 5, 6, 7, 8 and Fig. 2–3). These findings are consistent with previous studies on overall antibacterial consumption (Bindel and Seifert 2025b; ECDC 2024a), rational versus irrational prescribing behaviour (ECDC 2024a; Karakonstantis and Kalemaki 2019; Spernovasilis and Tsioutis 2024) and general rational medicine use (Bindel and Seifert 2025c; Rotar et al. 2020; Tian et al. 2023). Despite some progress, improvements in the use of antibacterial drugs remain insufficient, as highlighted in recent reports (ECDC 2024a; island.is 2024). Many countries are unlikely to meet the EU ‘One Health’ targets for rational prescribing and reduced overall consumption (Bindel and Seifert 2025b) (Tables 10 and 11).

Both inappropriate drug selection and overprescribing contribute to increasing bacterial resistance. Countries with high'Watch'shares and excessive consumption, particularly in Southern and Eastern Europe, report increased bacterial resistance rates, while countries with rational prescribing practices and lower consumption, mainly in Northern Europe, exhibit lower resistance levels (ECDC 2024b). For example, in 2023, the resistance rate of *K. pneumoniae* to 3rd generation cephalosporins was around 5–10% in Northern versus exceeding 50% in many Southern European countries (ECDC 2024b). However, prescribing behaviour varies significantly within regions, likely influenced by the presence or absence of national programs promoting antibacterial stewardship (MacBride-Stewart et al. 2021; Bojanic et al. 2018; Fürst et al. 2015; Wojkoska-Mach et al. 2018). The worsening antibacterial resistance crisis is exacerbated by the fact that high resistance rates render first-line antibacterial drugs ineffective, increase dependence on'back-up'drugs and accelerate the development of resistance. To break this cycle, overall antibacterial consumption needs to be reduced and the use of ‘Reserve’ antibacterials needs to be more tightly controlled in order to preserve the long-term efficacy of available treatments. Harmonised national policies, supported by universally accepted guidelines such as the ‘WHO Antibiotic Book’ (WHO 2022) and updates to the AWaRe framework to address classification gaps are essential for improving antibacterial use.

Further research is needed to refine these findings, explore additional drivers and extend the analysis to other regions, medicine classes and sectors of the ‘One Health’ approach. Predicting bacterial resistance trends alongside provides valuable insights for achieving the ‘One Health’ goals. Examining the prescribing patterns of specific antibacterial classes and substances would highlight critical trends, while detailed country-level assessments could inform targeted interventions to promote rational prescribing and achieve sustainable reductions in antibacterial consumption.

Proposals for action: Strengthen efforts to improve antibacterial stewardship.

### Practicing physicians: as little of the right as possible

1. **Prioritise strict rational prescribing practices:** Avoid empirical prescribing wherever possible, use narrow-spectrum antibacterials and adhere strictly to guidelines (provided by national authorities or the latest version of the “WHO AWaRe antibiotic book” https://iris.who.int/bitstream/handle/10665/365135/WHO-MHP-HPS-EML-2022.02-eng.pdf?sequence=1).
2. **Stay up to date:** Inform about changes in guidelines and adapt therapies to regional bacterial resistance patterns (provided by national authorities or the ECDC Surveillance Atlas of Infectious Diseases: https://atlas.ecdc.europa.eu/public/index.aspx?Dataset=27&HealthTopic=4).

### National health authorities: support rational prescribing behaviour

3. **Provide guidance:** Develop, publish and frequently update comprehensive guidelines on the use of antibacterial drugs.
4. **Restrict antibacterial drug use:** Enforce stricter regulations to control inappropriate usage, limit access to last-option antibacterials to critical cases, minimise over-the-counter sales and implement measures to ensure rational prescribing.
5. **Prevent supply shortages:** Ensure the availability of essential and widely used substances to prevent inappropriate reliance on back-up drugs to prevent increasing bacterial resistance and treatment failures.
6. **Monitor the consumption of antibacterial drugs and bacterial resistance trends:** Establish robust systems to collect and analyse data on antibacterial drug use and resistance patterns. Comprehensive datasets are essential to identify problematic developments and implement targeted interventions.

### Global health policy: support and coordinate national efforts

7. **Adopt common standards, targets and regulations:** Bacterial resistance transcends national borders. Establish unified strategies with clear targets to improve antibacterial stewardship across regions.
8. **Coordinate surveillance and refine classification systems:** Facilitate international cooperation to identify emerging resistance trends and improve global classification frameworks, such as the AWaRe system, to reduce gaps and inconsistencies in categorisation.
9. **Support and fund research**: Provide resources for the discovery of new antibacterial drugs. Create accessible and comprehensive datasets to enable thorough assessments of antibacterial consumption patterns and resistance trends.

## Supplementary Information

Below is the link to the electronic supplementary material.

Supplementary file1 (DOCX 106 KB)

## Data Availability

All source data for this study are available upon reasonable request from the authors.

## Abbreviations

ACF: Autocorrelation function
ARIMA: Auto Regressive Integrated Moving Average model (mathematical model for time series analysis)
AMC: Antimicrobial consumption
AMR: Antimicrobial resistance
AWaRe: Classification of antibacterial substances to support rational use and monitoring antibacterial consumption (divided into groups ‘Access’, ‘Watch’, ‘Reserve’) (WHO 2023)
DDD: Defined Daily Dose
DID: Defined Daily Dose prescriptions per 1000 inhabitants per day
ECDC: European Centre for Disease Prevention and Control (public health agency of the EU)
EU: European Union
LCL: Lower confidence limit
PACF: Partial autocorrelation function
SPSS: Statistical Product and Service Solutions (software for statistical analyses, IBM)
UCL: Upper confidence limit
WHO: World Health Organization
