# Supplementary material for: AWaRe classification analysis for European countries with ARIMA forecasts to assess prescribing patterns and ‘One Health’ targets
Source: Naunyn Schmiedebergs Arch Pharmacol. 2025 Apr 12;398(10):13707–29. doi: 10.1007/s00210-025-04121-y (PMC12511250; doi:10.1007/s00210-025-04121-y)
Supplement: Supplementary file 1 — Supplementary file1 (DOCX 106 KB) [file 210_2025_4121_MOESM1_ESM.docx]

**AWaRe classification analysis for European countries with ARIMA forecasts to assess prescribing patterns and ‘One Health’ targets**

**Lilly Josephine Bindel and Roland Seifert**

Supplemental Discussion

**Outstanding countries: confirmation of prudent and problematic antibacterial drug use patterns in recent years**

The proportions of antibacterial drug classes within the WHO AWaRe framework vary considerably between European countries, covering a wide spectrum from low to high proportions. However, consistent patterns emerge in which most AWaRe group shares tend to be similarly classified as good, moderate or poor (Table 2 and 4-6). To confirm this assessment of prudent and problematic drug use, a closer examination of the outstanding countries is necessary. In addition, certain exceptions deviate from these trends and require further discussion.

The 'Access' group is a critical indicator of prudent drug use. In 2023, Iceland (82.1%) and Denmark (80.1%) reported the highest proportions, both exceeding 80%, whereas Slovakia (41.7%), Bulgaria (42.0%), and Greece (42.0%) reported the lowest shares (Table 2). The 'Watch' group, which includes antibiotics recommended only for severe infections, serves as an indicator of potentially irrational antibacterial drug use. High proportions in this category are undesirable and were most pronounced in Slovakia (57.9%), Bulgaria (57.8%), and Greece (56.9%), whereas Iceland (13.7%) and Norway (14.4%) reported the lowest shares (Table 3). The 'Reserve' group, which comprises last-line treatments for serious infections, also exhibits a preference for lower proportions. While most countries reported shares between 0.0% and 0.5%, Greece (1.1%) and Austria (1.0%) stood out with higher values (Table 4). The 'Unclassified' group underscores the limitations of the current WHO classification system. While low shares are typically unremarkable, the exceptionally high proportions observed in Norway (24.0%) and Slovenia (10.9%) (Table 5) suggest potential gaps in the classification, though these figures do not directly indicate whether usage is rational or problematic.

Emerging patterns suggest that countries that stand out in one category often show similar trends in others (Figure 2), highlighting the interdependence of these shares. For example, Slovakia, Bulgaria and Greece not only have the lowest shares in the 'Access' group, but also the highest shares in the 'Watch' category. In addition, Greece stands out with a share above average in the 'Reserve' group, indicating additional concerns about antibacterial misuse. On the other hand, Iceland stands out for its exceptionally high 'Access' share and low 'Watch' share, an example of prudent antibacterial use. Norway, despite a low 'Watch' share, has a disproportionately high 'Unclassified' share, which complicates the overall assessment of its prescribing practices.

There is evidence of irrational use of antibacterial drugs in Greece, Bulgaria and Slovakia (Table 3-5). This conclusion is consistent with reports that south-eastern European countries in general have high antibacterial consumption and high rates of bacterial resistance (ECDC 2024a; Benko et al. 2022; Spernovasilis and Tsioutis 2024). The well-established link between high consumption and increasing bacterial resistance (Karakonstantis and Kalemaki 2019; Abejew et al. 2024; Olesen et al. 2018) further reinforces the concern. As resistance increases, many commonly used antibacterials lose their efficacy, leading to increased reliance on back-up drugs and exacerbating the resistance problem. In these regions, irrational prescribing behaviour is also influenced by factors such as cultural influences, insufficient knowledge and inadequate regulatory frameworks (Bindel and Seifert 2024c, 2025; Karakonstantis and Kalemaki 2019; Spernovasilis and Tsioutis 2024; Adekoya et al. 2021).

In contrast, Iceland, Denmark and Norway demonstrate commendable stewardship, as evidenced by their high shares in the 'Access' group and low shares in the 'Watch' group (Table 2 and 3). These countries maintain moderate or low overall consumption levels and low bacterial resistance rates (ECDC 2024b). Their success can be attributed to long-standing national action plans that promote rational use through clinical guidelines, restrictive policies and educational initiatives (Ministry of Health Iceland 2024; Ministry of Health Denmark 2017; Gutema et al. 2021).

Austria presents a unique case as it reports a relatively high 'Reserve' share despite maintaining average shares in other categories (Table 3-5). This discrepancy may be tempered by the fact that Austria has the lowest total antibacterial consumption in 2023 (Bindel and Seifert 2025b). However, this high proportion still suggests a less critical approach to last-resort use, which needs to be further optimised. Another country with an alarmingly high 'Reserve' share is Germany (Table 5). In contrast to Austria, this problematic situation is also reflected in the highest consumption of 'Reserve' substances among the countries analysed and is exaggerated by the fact that only consumption in the community sector is covered (Table S1), whereas severe infections are most likely to be treated in hospitals. As the proportions of 'Access' and 'Watch' are also suboptimal (Table 2 and 4), this reinforces the consideration of a more irrational use, which is confirmed by previous research (Bindel and Seifert 2024c).

The unusually high consumption recorded in the 'Unclassified' group for Norway and Slovenia (Table 6) raises concerns about the completeness of the AWaRe classification, but also helps to explain discrepancies, such as a moderate level of 'Access' alongside an exceptionally low proportion of 'Watch' (Table 2 and 4). The lack of detailed information on substances in this category makes it difficult to assess prescribing behaviour. If the 'Unclassified' group were to be reclassified into 'Access', this would likely confirm prudent antibacterial use in these countries. Refining the classification of unclassified substances within AWaRe would enable a more accurate assessment of prescribing practices.

In summary, the distribution of AWaRe groups among the outstanding countries reflects broader patterns of prescribing behaviour, overall consumption levels and bacterial resistance. The analysis of prescribing behaviour by the distribution of AWaRe groups in 2023 is confirmed. Greece, Bulgaria and Slovakia are assumed as having particularly problematic prescribing behaviour, and Germany stands out for its alarmingly high use of 'Reserve' substances. In contrast, Iceland, Norway and Denmark exhibit prudent use. The limitations of the AWaRe classification system for some countries highlight the need for further refinement to allow more accurate assessments, but also explain possible discrepancies in the distribution of AWaRe groups for certain countries.

**Geographical distribution of drug prescribing behaviour: regional patterns in recent and future trends**

Northern Europe is characterised by cautious use, as evidenced by a high proportion of the 'Access' group and low proportions of the 'Watch' and 'Reserve' groups, both in current figures and in projections for 2030 (Figure 2-3; Table 2-7 and 9). However, the disproportionately high share of 'Unclassified' antibacterials complicates interpretation, if these substances were not predominantly problematic, Northern Europe could be considered even more favourable.

Western Europe shows similarly positive patterns, with high proportions of the 'Access' group and low proportions of the 'Watch', 'Reserve' and 'Unclassified' groups. However, the presence of a comparatively high share of 'Unclassified' limits a full assessment, and projected trends suggest a worsening situation in the 'Access' and 'Watch' groups, despite improvements in the 'Reserve' and 'Unclassified' groups. Central Europe shows a moderate level of antibacterial use, with moderate proportions in both the 'Access' and 'Reserve' groups, and a pleasingly low proportion in the 'Watch' group. However, projections show a slightly worsening trend, characterised by a decrease in the 'Access' group and an increase in the 'Watch' group, although these changes remain within acceptable limits.

Eastern Europe currently shows problematic behaviour, characterised by a low proportion of the 'Access' group and a high proportion of the 'Watch' group, together with moderate use of the 'Reserve' group and low levels of 'Unclassified' substances. Although the forecasts predict improvements, with increasing proportions of the 'Access' group and decreasing proportions of the 'Watch' and 'Reserve' groups, overall consumption levels in Eastern Europe remain far from ideal and problematic practices persist.

Southern Europe presents the most worrying scenario, combining an already low share of the 'Access' group with high shares of both the 'Watch' and 'Reserve' groups, which are projected to deteriorate further by 2030. The 'Unclassified' group remains unremarkable in this region.

**Correlation between rational antibacterial drug use and total consumption volume**

For this analysis, results and projections from a previous analysis of antibacterial drug consumption in Europe were used (Bindel and Seifert 2025b). A bivariate correlation analysis was carried out between the share and the consumption of the ‘Access’ group as well as between the share of the ‘Access’ group and the total consumption volume in 2023 (Table 10, Figure 4).

A significant negative correlation with a correlation coefficient of -0.415 was observed between the ‘Access’ group share and total antibacterial consumption in 2023 (Table 12). This result implies that higher ‘Access’ group shares are associated with lower total consumption volumes (Figure 4). This relationship is observed in many countries. For example, the Netherlands, Latvia, Finland and Sweden have relatively low consumption volumes and high shares of the ‘Access’ group. On the other hand, Bulgaria, Greece, Romania, Cyprus or Slovakia have high consumption volumes and low shares. However, there are also countries with discrepancies, such as Belgium and France with a high share of the ‘Access’ group but also a comparatively high consumption. This suggests that the two factors are related, but that there may be differences. One reason for this may be that some countries have already achieved more rational use of antibacterial drugs, such as Northern European countries, while there are countries with very problematic use, such as South-Eastern countries.

In contrast, there is no significant correlation between the share and the consumption of the ‘Access’ group in 2023 (Table 12). This can be explained by the fact that there are countries with a high share but generally low consumption, while other countries have a low share but high consumption (Figure 4). Different variations are therefore possible, leading to a non-significant result.

In summary, there is a significant correlation between the cautious use of antibacterial drugs (indicated by the share of the ‘Access’ group) and the total volume of consumption, but not between the share and the consumption within the ‘Access’ group. It makes sense that the DID of a particular sector would depend on the total consumption rather than the proportion within it, since the consumption of an individual sector is determined by the total consumption. This relationship can be seen as a general trend, with variations in manifestation within individual countries (Figure 4), and highlights the potential of the ‘Access’ group share as a measure of prudent antibacterial use, reflecting both rational treatment choice and low consumption. In many cases, if one target of the ‘One Health’ approach for the human sector is projected to be achieved, the other could also be reached. While the combination of ‘Access’ rate and prescription volume is a good indicator of the rational use of antibacterial drugs, the ‘One Health’ approach combines a fortunate level with continuing improvement, the possibility of improvement or an uncertain development.

Supplemental Tables

***Table S1:*** *Data availability for analysed countries and assessment of data quality for the share of the group ‘Access’ (good in green, moderate in yellow, poor in orange). Furthermore, information about covered sectors and population (ESAC-net 2025) is provided. Published data for the other AWaRe groups ‘Watch’, ‘Reserve’ and ‘Unclassified’, as well as consumption volumes in DID may vary slightly.*

| **country** | **'Access' group share years with published data** | **Assessment of data quality for the 'Access' group** | **Information about data methodology (ESAC-net 2025)** | | |
| --- | --- | --- | --- | --- | --- |
|  |  |  | **covered health care sectors (community, hospital)** | **origin of consumption data (reimbursement, sales)** | **population coverage** |
| **Austria** | 2019-2023 | poor | both | reimbursement (community); sales (hospital) | 100% |
| **Belgium** | 1997-2023 | good | both | reimbursement | 99% (community); 81% (hospital) |
| **Bulgaria** | 1999-2023 | good | both | sales | 100% |
| **Croatia** | 2000-2023 | good | both | reimbursement | 100% |
| **Cyprus** | 2006-2022 | moderate | both | sales | 100% |
| **Czechia** | 2019-2023 | poor | both | both | 100% |
| **Denmark** | 1997-2023 | good | both | sales | 100% |
| **Estonia** | 2001-2023 | good | both | sales | 100% |
| **Finland** | 1997-2023 | good | both | sales | 100% |
| **France** | 1997-2023 | good | both | sales | 100% |
| **Germany** | 2002-2004, 2023 | poor | community only | reimbursement (community) | 88% |
| **Greece** | 1997-2023 | good | both | both (community); sales (hospital) | 100% |
| **Hungary** | 2001-2023 | good | both | sales | 100% (community); 99% (hospital) |
| **Iceland** | 1997-2005, 2010-2013, 2017-2023 | moderate | both | sales | 100% (community); 99% (hospital) |
| **Ireland** | 2004-2023 | moderate | both | both (community); sales (hospital) | 100% |
| **Italy** | 2005, 2007-2008, 2010-2023 | moderate | both | both (community); reimbursement (hospital) | 100% |
| **Latvia** | 2002, 2004-2023 | moderate | both | sales | 100% |
| **Lithuania** | 2006-2023 | moderate | both | sales | 100% |
| **Luxembourg** | 1997-2023 | good | both | reimbursement (community); sales (hospital) | 100%; 90% |
| **Malta** | 2007-2023 | moderate | both | sales | 100% |
| **Netherlands** | 1997-2002, 2010-2023 | moderate | both | sales | 93% (community); 94% (hospital) |
| **Norway** | 1998, 2001-2023 | good | both | both | 100% |
| **Poland** | 1998-2002, 2004, 2014-2023 | moderate | both | not mentioned | 100% |
| **Portugal** | 2009-2023 | moderate | both | reimbursement (community); both (hospital) | 100% (community); 75% (hospital) |
| **Romania** | 2009, 2011-2023 | moderate | both | sales (community); reimbursement (hospital) | 90% (community); 100% (hospital) |
| **Slovakia** | 1999-2009, 2011-2023 | good | both | sales | 100% |
| **Slovenia** | 1997-2023 | good | both | sales | 100% |
| **Spain** | 2016-2023 | poor | both | sales | 100% |
| **Sweden** | 1997-2021 | good | both | sales | 100% |
| **United Kingdom** | 2013-2019 | poor | both | reimbursement (community); sales (hospital) | 100% |

***Table S2:*** *Python Code for the ADF test and estimation of optimal parameters for individual countries.*

!pip install pandas pmdarima openpyxl statsmodels

import pandas as pd

from pmdarima import auto_arima

from statsmodels.tsa.stattools import adfuller

from google.colab import files

# Step 1: Upload the file

print("Please upload your Excel file:")

uploaded = files.upload()

# Check the name of the uploaded file

file_path = list(uploaded.keys())[0]

print(f"File '{file_path}' successfully uploaded.")

# Step 2: Load the Excel file into a DataFrame

df = pd.read_excel(file_path, sheet_name=0) # Load the first sheet

# Step 3: Verify the "Time period" column and set as index

if 'Time period' in df.columns:

print("'Time period' column found. Here are the first few values:")

print(df['Time period'].head())

df.set_index('Time period', inplace=True)

else:

raise ValueError("The column 'Time period' is missing. Please check your file.")

# Step 4: Iterate through each time series (country)

results = []

excluded_countries = []

for country in df.columns:

series = df[country]

# Check if enough data points are available for analysis (at least 3)

if len(series.dropna()) < 3:

excluded_countries.append(country)

continue

# Step 5: Perform ADF test for d=0, d=1, and d=2 and store results

try:

adf_result_d0 = adfuller(series.dropna())

adf_statistic_d0 = adf_result_d0[0]

p_value_d0 = adf_result_d0[1]

except ValueError:

adf_statistic_d0 = None

p_value_d0 = None

try:

adf_result_d1 = adfuller(series.diff().dropna())

adf_statistic_d1 = adf_result_d1[0]

p_value_d1 = adf_result_d1[1]

except ValueError:

adf_statistic_d1 = None

p_value_d1 = None

try:

adf_result_d2 = adfuller(series.diff().diff().dropna())

adf_statistic_d2 = adf_result_d2[0]

p_value_d2 = adf_result_d2[1]

except ValueError:

adf_statistic_d2 = None

p_value_d2 = None

# Determine differencing order (d) based on ADF tests

if p_value_d0 is not None and p_value_d0 <= 0.05:

d = 0

elif p_value_d1 is not None and p_value_d1 <= 0.05:

d = 1

elif p_value_d2 is not None and p_value_d2 <= 0.05:

d = 2

else:

d = 0 # Fallback to d=0 if none are stationary

# Step 6: Find optimal ARIMA parameters using BIC and determined d

model = auto_arima(

series.dropna(),

seasonal=False,

stepwise=True,

suppress_warnings=True,

trace=True, # You might want to set this to False for cleaner output

error_action='ignore',

information_criterion='bic',

d=d,

max_d=2

)

# Store results, including ADF test results for d=0, d=1, d=2

results.append({

'Country': country,

'p': model.order[0],

'd': model.order[1],

'q': model.order[2],

'AIC': model.aic(),

'BIC': model.bic(),

'ADF Statistic (d=0)': adf_statistic_d0,

'ADF p-value (d=0)': p_value_d0,

'ADF Statistic (d=1)': adf_statistic_d1,

'ADF p-value (d=1)': p_value_d1,

'ADF Statistic (d=2)': adf_statistic_d2,

'ADF p-value (d=2)': p_value_d2

})

# Step 7: Create and save results DataFrame

results_df = pd.DataFrame(results)

results_df.to_excel("individual_arima_parameters_with_adf_all_d.xlsx", index=False)

***Table S3:*** *Results from the Python Code of optimal ARIMA parameters for individual countries (see Table S2).*

| **Country** | **p** | **d** | **q** | **AIC** | **BIC** | **ADF Statistic (d=0)** | **ADF p-value (d=0)** | **ADF Statistic (d=1)** | **ADF p-value (d=1)** | **ADF Statistic (d=2)** | **ADF p-value (d=2)** |
| --- | --- | --- | --- | --- | --- | --- | --- | --- | --- | --- | --- |
| Austria | 1 | 0 | 1 | -26.462 | -28.025 | -3.019 | 0.033 | -2.053 | 0.264 |  |  |
| Belgium | 0 | 1 | 0 | -142.180 | -140.922 | -0.889 | 0.792 | -4.337 | 0.000 | -2.654 | 0.082 |
| Bulgaria | 0 | 2 | 2 | -62.374 | -57.832 | -1.880 | 0.341 | -0.234 | 0.934 | -3.497 | 0.008 |
| Croatia | 0 | 1 | 0 | -126.641 | -125.506 | -1.455 | 0.556 | -2.883 | 0.047 | -1.097 | 0.716 |
| Cyprus | 0 | 1 | 0 | -66.180 | -65.407 | 0.018 | 0.960 | -12.392 | 0.000 | -2.891 | 0.046 |
| Czechia | 0 | 2 | 0 | -10.710 | -11.611 | -1.670 | 0.447 | -1.736 | 0.412 |  |  |
| Denmark | 0 | 1 | 0 | -176.724 | -175.466 | -1.117 | 0.708 | -4.255 | 0.001 | -3.796 | 0.003 |
| Estonia | 1 | 0 | 3 | -112.476 | -105.663 | -2.938 | 0.041 | -2.512 | 0.113 | 0.514 | 0.985 |
| Finland | 0 | 1 | 0 | -151.475 | -150.217 | -1.775 | 0.393 | -5.155 | 0.000 | -10.508 | 0.000 |
| France | 1 | 1 | 0 | -133.160 | -129.386 | -0.587 | 0.874 | -4.062 | 0.001 | -8.728 | 0.000 |
| Germany | 0 | 0 | 0 | -12.689 | -13.916 | -2.257 | 0.186 |  |  |  |  |
| Greece | 0 | 1 | 0 | -101.119 | -99.861 | -2.736 | 0.068 | -5.727 | 0.000 | -5.997 | 0.000 |
| Hungary | 0 | 2 | 2 | -98.773 | -94.595 | -1.952 | 0.308 | 1.839 | 0.998 | -2.120 | 0.237 |
| Iceland | 0 | 1 | 0 | -114.033 | -113.089 | 0.593 | 0.987 | -4.339 | 0.000 | -2.461 | 0.125 |
| Ireland | 2 | 2 | 2 | -95.544 | -90.202 | 5.207 | 1.000 | -0.143 | 0.945 | -2.831 | 0.054 |
| Italy | 0 | 1 | 1 | -81.797 | -80.252 | -2.426 | 0.135 | -5.292 | 0.000 | -2.339 | 0.160 |
| Latvia | 0 | 2 | 1 | -93.919 | -91.086 | -1.192 | 0.677 | -0.182 | 0.941 | -1.450 | 0.558 |
| Lithuania | 0 | 2 | 0 | -77.810 | -77.037 | -2.475 | 0.122 | -2.463 | 0.125 | -3.905 | 0.002 |
| Luxembourg | 0 | 1 | 0 | -113.745 | -112.487 | -1.248 | 0.653 | -5.455 | 0.000 | -3.608 | 0.006 |
| Malta | 0 | 2 | 1 | -51.777 | -50.361 | -0.654 | 0.858 | -1.818 | 0.372 | -5.688 | 0.000 |
| Netherlands | 0 | 1 | 0 | -134.138 | -133.194 | -1.201 | 0.673 | -11.661 | 0.000 | -4.401 | 0.000 |
| Norway | 1 | 0 | 0 | -116.598 | -113.064 | -3.297 | 0.015 | -4.772 | 0.000 | -2.863 | 0.050 |
| Poland | 1 | 0 | 0 | -56.214 | -53.896 | -12.234 | 0.000 | -3.968 | 0.002 | -3.199 | 0.020 |
| Portugal | 2 | 0 | 0 | -38.887 | -36.763 | -12.202 | 0.000 | -1.681 | 0.441 | -35.097 | 0.000 |
| Romania | 0 | 1 | 0 | -42.058 | -41.493 | -2.208 | 0.203 | -6.631 | 0.000 | -0.058 | 0.953 |
| Slovakia | 0 | 2 | 1 | -82.885 | -80.703 | -2.351 | 0.156 | -1.598 | 0.485 | -4.226 | 0.001 |
| Slovenia | 0 | 1 | 0 | -109.105 | -107.847 | -2.668 | 0.080 | -5.590 | 0.000 | -8.322 | 0.000 |
| Spain | 1 | 1 | 0 | -55.640 | -55.802 | 0.760 | 0.991 | -9.798 | 0.000 | -11.318 | 0.000 |
| Sweden | 0 | 1 | 0 | -141.998 | -140.820 | -2.050 | 0.265 | -4.700 | 0.000 | -3.977 | 0.002 |
| United Kingdom | 1 | 2 | 0 | -39.210 | -40.382 | 4.974 | 1.000 | 5.601 | 1.000 | -3.910 | 0.002 |

***Table S4:*** *Python code and output for ADF test and estimation of generalised optimal parameters for all countries.*

!pip install pandas pmdarima openpyxl statsmodels

import pandas as pd

from pmdarima import auto_arima

from statsmodels.tsa.stattools import adfuller

from google.colab import files

# Step 1: Upload the file

print("Please upload your Excel file:")

uploaded = files.upload()

# Check the name of the uploaded file

file_path = list(uploaded.keys())[0]

print(f"File '{file_path}' successfully uploaded.")

# Step 2: Load the Excel file into a DataFrame

df = pd.read_excel(file_path, sheet_name=0) # Load the first sheet

# Step 3: Verify the "Time period" column and set as index

if 'Time period' in df.columns:

print("'Time period' column found. Here are the first few values:")

print(df['Time period'].head())

df.set_index('Time period', inplace=True)

else:

raise ValueError("The column 'Time period' is missing. Please check your file.")

# Step 4: Combine all time series into one

all_series = df.values.ravel() # Combine all data into a 1D array

all_series = pd.Series(all_series) # Convert to pandas Series

# Step 5: Perform ADF test and determine differencing order (up to d=2)

try:

adf_result = adfuller(all_series.dropna())

p_value = adf_result[1]

if p_value > 0.05:

diff_series = all_series.diff().dropna()

if len(diff_series) > 1:

try:

adf_result_diff1 = adfuller(diff_series)

p_value_diff1 = adf_result_diff1[1]

if p_value_diff1 > 0.05:

diff_series2 = diff_series.diff().dropna()

if len(diff_series2) > 1:

d = 2

else:

d = 1

else:

d = 1

except ValueError:

d = 0

else:

d = 0

else:

d = 0

except ValueError:

d = 0

# Step 6: Find optimal ARIMA parameters using BIC and determined d

model = auto_arima(

all_series.dropna(),

seasonal=False,

stepwise=True,

suppress_warnings=True,

trace=True, # You might want to set this to False for cleaner output

error_action='ignore',

information_criterion='bic',

d=d,

max_d=2

)

# Print the best model parameters

print("Best model:", model)

# Extract and save the parameters

p, d, q = model.order

with open("global_arima_parameters.txt", "w") as f:

f.write(f"p: {p}\n")

f.write(f"d: {d}\n")

f.write(f"q: {q}\n")

print("Global ARIMA parameters saved to global_arima_parameters.txt")

**Output**

**Best model: ARIMA(1,0,1)(0,0,0)[0] Total fit time: 4.376 seconds**

***Table S5:*** *Fit metrics for individual and generalized ARIMA models with optimal parameters for the ‘Access’ group without outlier detection.* *A good fit in green colour is considered to be stationary R-squared above 0.65, R-squared above 0.85, MAPE below 6 and MaxAPE below 15. Moderate fit in yellow colour includes R-squared between 0.4 and 0.65, R-squared between 0.6 and 0.84, MAPE between 7 and 20 and MaxAPE between 16 and 40. Poor fit in orange colour is indicated by R-squared less than 0.4, R-squared less than 0.6, MAPE greater than 20 and MaxAPE greater than 40.*

| **Country** | **Model** | **Stationary R-squared** | **R-squared** | **RMSE** | **MAPE** | **MaxAPE** | **MAE** | **MaxAE** | **Normalized BIC** |
| --- | --- | --- | --- | --- | --- | --- | --- | --- | --- |
| **Austria** | ARIMA(1,0,1) | 0.28 | 0.28 | 0.016 | 1.382 | 0.008 | 3.254 | 0.019 | -7.318 |
|  | - |  |  |  |  |  |  |  |  |
| **Belgium** | ARIMA(0,1,0) | 0 | 0.919 | 0.015 | 2.071 | 0.013 | 5.421 | 0.031 | -8.285 |
|  | ARIMA(1,0,1) | 0.904 | 0.904 | 0.017 | 2.057 | 0.012 | 6.297 | 0.037 | -7.833 |
| **Bulgaria** | ARIMA(0,2,2) | 0.448 | 0.766 | 0.067 | 6.669 | 0.041 | 27.629 | 0.165 | -4.992 |
|  | ARIMA(1,0,1) | 0.725 | 0.725 | 0.079 | 8.023 | 0.05 | 29.63 | 0.248 | -4.681 |
| **Croatia** | ARIMA(0,1,0) | -4.44E-16 | 0.689 | 0.015 | 2.003 | 0.012 | 4.527 | 0.027 | -8.258 |
|  | ARIMA(1,0,1) | 0.716 | 0.716 | 0.015 | 1.94 | 0.012 | 4.309 | 0.025 | -8.006 |
| **Cyprus** | ARIMA(0,1,0) | -8.88E-16 | 0.02 | 0.03 | 4.318 | 0.022 | 12.333 | 0.068 | -6.871 |
|  | ARIMA(1,0,1) | 0.37 | 0.37 | 0.025 | 3.471 | 0.017 | 13.499 | 0.06 | -6.918 |
| **Czechia** | ARIMA(0,2,0) | -2.22E-16 | -7.246 | 0.036 | 4.533 | 0.027 | 6.781 | 0.041 | -6.303 |
|  | ARIMA(1,0,1) | 0.249 | 0.249 | 0.014 | 1.368 | 0.008 | 1.918 | 0.012 | -7.555 |
| **Denmark** | ARIMA(0,1,0) | -2.22E-16 | 0.835 | 0.007 | 0.758 | 0.006 | 2.458 | 0.019 | -9.663 |
|  | ARIMA(1,0,1) | 0.731 | 0.731 | 0.01 | 0.886 | 0.007 | 4.733 | 0.035 | -8.759 |
| **Estonia** | ARIMA(1,0,3) | 0.834 | 0.834 | 0.034 | 2.271 | 0.016 | 15.213 | 0.125 | -6.098 |
|  | ARIMA(1,0,1) | 0.84 | 0.84 | 0.031 | 2.507 | 0.017 | 13.402 | 0.11 | -6.511 |
| **Finland** | ARIMA(0,1,0) | 0 | 0.531 | 0.013 | 1.403 | 0.01 | 4.329 | 0.03 | -8.576 |
|  | ARIMA(1,0,1) | 0.562 | 0.562 | 0.014 | 1.438 | 0.01 | 4.744 | 0.032 | -8.222 |
| **France** | ARIMA(1,1,0) | 0.156 | 0.934 | 0.017 | 2.276 | 0.014 | 8.341 | 0.044 | -7.851 |
|  | ARIMA(1,0,1) | 0.893 | 0.893 | 0.022 | 2.808 | 0.017 | 9.296 | 0.055 | -7.244 |
| **Germany** | ARIMA(0,0,0) | 5.96E-14 | 5.96E-14 | 0.035 | 4.543 | 0.027 | 6.907 | 0.044 | -6.376 |
|  | ARIMA(1,0,1) | 0.149 | 0.149 | 0.055 | 3.723 | 0.022 | 7.215 | 0.046 | -4.745 |
| **Greece** | ARIMA(0,1,0) | -8.88E-16 | 0.566 | 0.033 | 5.833 | 0.025 | 20.748 | 0.088 | -6.667 |
|  | ARIMA(1,0,1) | 0.544 | 0.544 | 0.04 | 6.452 | 0.028 | 20.496 | 0.115 | -6.074 |
| **Hungary** | ARIMA(0,2,2) | 0.546 | 0.743 | 0.023 | 3.022 | 0.016 | 8.904 | 0.053 | -7.108 |
|  | ARIMA(1,0,1) | 0.723 | 0.723 | 0.027 | 3.337 | 0.018 | 12.264 | 0.077 | -6.793 |
| **Iceland** | ARIMA(0,1,0) | 2.22E-16 | 0.502 | 0.011 | 0.914 | 0.008 | 3.572 | 0.031 | -8.786 |
|  | ARIMA(1,0,1) | 0.487 | 0.487 | 0.012 | 0.997 | 0.008 | 3.645 | 0.032 | -8.323 |
| **Ireland** | ARIMA(2,2,2) | 0.652 | 0.95 | 0.016 | 1.661 | 0.01 | 6.855 | 0.039 | -7.419 |
|  | ARIMA(1,0,1) | 0.846 | 0.846 | 0.027 | 2.731 | 0.017 | 14.306 | 0.082 | -6.808 |
| **Italy** | ARIMA(0,1,1) | 0.341 | -0.4 | 0.018 | 2.336 | 0.011 | 10.997 | 0.049 | -7.722 |
|  | ARIMA(1,0,1) | 0.03 | 0.03 | 0.017 | 2.28 | 0.011 | 7.091 | 0.036 | -7.701 |
| **Latvia** | ARIMA(0,2,1) | 0.512 | 0.499 | 0.019 | 2.098 | 0.015 | 5.194 | 0.037 | -7.62 |
|  | ARIMA(1,0,1) | 0.63 | 0.63 | 0.029 | 2.58 | 0.019 | 10.38 | 0.089 | -6.637 |
| **Lithuania** | ARIMA(0,2,0) | 0 | 0.533 | 0.021 | 2.123 | 0.015 | 6.386 | 0.043 | -7.588 |
|  | ARIMA(1,0,1) | 0.591 | 0.591 | 0.032 | 2.592 | 0.019 | 11.769 | 0.099 | -6.395 |
| **Luxembourg** | ARIMA(0,1,0) | 0 | 0.552 | 0.027 | 2.625 | 0.014 | 16.968 | 0.098 | -7.131 |
|  | ARIMA(1,0,1) | 0.581 | 0.581 | 0.026 | 2.969 | 0.016 | 14.588 | 0.084 | -6.9 |
| **Malta** | ARIMA(0,2,1) | 0.268 | 0.622 | 0.04 | 6.4 | 0.03 | 15.736 | 0.07 | -6.088 |
|  | ARIMA(1,0,1) | 0.707 | 0.707 | 0.035 | 5.666 | 0.026 | 17.477 | 0.073 | -6.219 |
| **Netherlands** | ARIMA(0,1,0) | 0 | 0.773 | 0.006 | 0.727 | 0.005 | 1.799 | 0.013 | -9.947 |
|  | ARIMA(1,0,1) | 0.731 | 0.731 | 0.007 | 0.778 | 0.005 | 2.025 | 0.015 | -9.37 |
| **Norway** | ARIMA(1,0,0) | 0.633 | 0.633 | 0.024 | 2.219 | 0.015 | 10.608 | 0.079 | -7.166 |
|  | ARIMA(1,0,1) | 0.654 | 0.654 | 0.024 | 2.351 | 0.015 | 10.09 | 0.075 | -7.046 |
| **Poland** | ARIMA(1,0,0) | 0.738 | 0.738 | 0.037 | 4.751 | 0.031 | 8.891 | 0.064 | -6.233 |
|  | ARIMA(1,0,1) | 0.745 | 0.745 | 0.038 | 4.721 | 0.031 | 8.897 | 0.064 | -6.011 |
| **Portugal** | ARIMA(2,0,0) | -0.116 | -0.116 | 0.074 | 7.603 | 0.038 | 44.438 | 0.171 | -4.654 |
|  | ARIMA(1,0,1) | -0.073 | -0.073 | 0.073 | 7.712 | 0.038 | 47.09 | 0.173 | -4.693 |
| **Romania** | ARIMA(0,1,0) | 1.11E-15 | 0.808 | 0.017 | 2.42 | 0.013 | 5.37 | 0.031 | -7.909 |
|  | ARIMA(1,0,1) | 0.286 | 0.286 | 0.043 | 4.095 | 0.023 | 18.348 | 0.115 | -5.747 |
| **Slovakia** | ARIMA(0,2,1) | 0.39 | 0.879 | 0.04 | 7.024 | 0.033 | 18.758 | 0.087 | -6.145 |
|  | ARIMA(1,0,1) | 0.847 | 0.847 | 0.053 | 6.446 | 0.033 | 24.568 | 0.185 | -5.479 |
| **Slovenia** | ARIMA(0,1,0) | -4.44E-16 | 0.13 | 0.029 | 2.792 | 0.017 | 21.417 | 0.122 | -6.95 |
|  | ARIMA(1,0,1) | 0.318 | 0.318 | 0.027 | 2.692 | 0.017 | 17.29 | 0.099 | -6.867 |
| **Spain** | ARIMA(1,1,0) | 0.845 | 0.823 | 0.003 | 0.294 | 0.002 | 0.901 | 0.006 | -11.013 |
|  | ARIMA(1,0,1) | 0.181 | 0.181 | 0.008 | 0.708 | 0.004 | 1.925 | 0.012 | -9.003 |
| **Sweden** | ARIMA(0,1,0) | -2.22E-16 | 0.39 | 0.012 | 1.23 | 0.009 | 4.262 | 0.031 | -8.698 |
|  | ARIMA(1,0,1) | 0.451 | 0.451 | 0.013 | 1.312 | 0.009 | 3.983 | 0.028 | -8.368 |
| **United Kingdom** | ARIMA(1,2,0) | 0.42 | 0.923 | 0.004 | 0.383 | 0.003 | 0.573 | 0.004 | -10.66 |
|  | ARIMA(1,0,1) | 0.532 | 0.532 | 0.009 | 0.823 | 0.005 | 2.245 | 0.015 | -8.489 |

***Table S6:*** *Fit metrics for generalized ARIMA models with optimal parameters for the ‘Watch’ group.* *A good fit in green colour is considered to be stationary R-squared above 0.65, R-squared above 0.85, MAPE below 6 and MaxAPE below 15. Moderate fit in yellow colour includes R-squared between 0.4 and 0.65, R-squared between 0.6 and 0.84, MAPE between 7 and 20 and MaxAPE between 16 and 40. Poor fit in orange colour is indicated by R-squared less than 0.4, R-squared less than 0.6, MAPE greater than 20 and MaxAPE greater than 40.*

| **Model Statistics for the 'Watch' group with ARIMA(1,0,1) without outlier detection** | | | | | | | | |
| --- | --- | --- | --- | --- | --- | --- | --- | --- |
| **Country** | **Stationary R-squared** | **R-squared** | **RMSE** | **MAPE** | **MAE** | **MaxAPE** | **MaxAE** | **Normalized BIC** |
| **Austria** | 0.102 | 0.102 | 0.016 | 2.358 | 0.009 | 3.942 | 0.016 | -7.328 |
| **Belgium** | 0.865 | 0.865 | 0.018 | 3.565 | 0.014 | 9.273 | 0.036 | -7.681 |
| **Bulgaria** | 0.723 | 0.723 | 0.091 | 33.74 | 0.051 | 547.738 | 0.299 | -4.41 |
| **Croatia** | 0.816 | 0.816 | 0.019 | 4.303 | 0.014 | 15.293 | 0.046 | -7.543 |
| **Cyprus** | 0.362 | 0.362 | 0.025 | 3.555 | 0.017 | 10.768 | 0.06 | -6.911 |
| **Czechia** | 0.209 | 0.209 | 0.013 | 1.867 | 0.007 | 2.765 | 0.01 | -7.759 |
| **Denmark** | 0.802 | 0.802 | 0.009 | 3.219 | 0.007 | 11.107 | 0.025 | -8.994 |
| **Estonia** | 0.838 | 0.838 | 0.032 | 6.547 | 0.017 | 61.745 | 0.11 | -6.494 |
| **Finland** | 0.370 | 0.37 | 0.01 | 3.167 | 0.007 | 15.79 | 0.028 | -8.82 |
| **France** | 0.909 | 0.909 | 0.018 | 4.193 | 0.015 | 10.684 | 0.04 | -7.626 |
| **Germany** | 0.207 | 0.207 | 0.043 | 5.84 | 0.02 | 8.073 | 0.027 | -5.244 |
| **Greece** | 0.551 | 0.551 | 0.041 | 5.38 | 0.029 | 27.974 | 0.119 | -6.012 |
| **Hungary** | 0.728 | 0.728 | 0.027 | 4.129 | 0.018 | 21.474 | 0.079 | -6.787 |
| **Iceland** | 0.212 | 0.212 | 0.012 | 6.579 | 0.008 | 32.834 | 0.032 | -8.41 |
| **Ireland** | 0.845 | 0.845 | 0.027 | 4.867 | 0.017 | 19.709 | 0.084 | -6.781 |
| **Italy** | 0.044 | 0.044 | 0.017 | 2.14 | 0.011 | 7.85 | 0.038 | -7.6 |
| **Latvia** | 0.639 | 0.639 | 0.03 | 9.006 | 0.02 | 64.735 | 0.091 | -6.611 |
| **Lithuania** | 0.607 | 0.607 | 0.032 | 8.365 | 0.019 | 63.246 | 0.099 | -6.423 |
| **Luxembourg** | 0.498 | 0.498 | 0.028 | 4.35 | 0.019 | 18.648 | 0.079 | -6.787 |
| **Malta** | 0.716 | 0.716 | 0.035 | 5.106 | 0.026 | 12.629 | 0.073 | -6.22 |
| **Netherlands** | 0.267 | 0.267 | 0.007 | 1.815 | 0.005 | 5.094 | 0.015 | -9.533 |
| **Norway** | 0.812 | 0.812 | 0.01 | 4.077 | 0.007 | 13.579 | 0.024 | -8.893 |
| **Poland** | 0.787 | 0.787 | 0.038 | 8.034 | 0.025 | 39.355 | 0.094 | -6.016 |
| **Portugal** | 0.001 | 0.001 | 0.031 | 5.782 | 0.022 | 21.955 | 0.07 | -6.376 |
| **Romania** | 0.281 | 0.281 | 0.042 | 5.333 | 0.023 | 30.567 | 0.114 | -5.752 |
| **Slovakia** | 0.842 | 0.842 | 0.054 | 8.487 | 0.034 | 77.195 | 0.188 | -5.455 |
| **Slovenia** | 0.808 | 0.808 | 0.016 | 4.031 | 0.013 | 9.601 | 0.032 | -7.879 |
| **Spain** | 0.068 | 0.068 | 0.007 | 1.086 | 0.004 | 2.978 | 0.011 | -9.262 |
| **Sweden** | 0.517 | 0.517 | 0.01 | 3.163 | 0.007 | 10.026 | 0.023 | -8.907 |
| **United Kingdom** | 0.613 | 0.613 | 0.009 | 1.478 | 0.005 | 4.154 | 0.014 | -8.569 |

***Table S7:*** *Fit metrics for generalized ARIMA models with optimal parameters for the ‘Reserve’ group.* *A good fit in green colour is considered to be stationary R-squared above 0.65, R-squared above 0.85, MAPE below 6 and MaxAPE below 15. Moderate fit in yellow colour includes R-squared between 0.4 and 0.65, R-squared between 0.6 and 0.84, MAPE between 7 and 20 and MaxAPE between 16 and 40. Poor fit in orange colour is indicated by R-squared less than 0.4, R-squared less than 0.6, MAPE greater than 20 and MaxAPE greater than 40.*

| **Model Statistics for the 'Reserve' group with ARIMA(1,0,1) without outlier detection** | | | | | | | | |
| --- | --- | --- | --- | --- | --- | --- | --- | --- |
| **Country** | **Stationary R-squared** | **R-squared** | **RMSE** | **MAPE** | **MAE** | **MaxAPE** | **MaxAE** | **Normalized BIC** |
| **Austria** | 0.154 | 0.154 | 0.002 | 11.127 | 0.001 | 31.023 | 0.003 | -11.256 |
| **Belgium** | 0.132 | 0.132 | 0.001 | 67.209 | 0.001 | 325.398 | 0.005 | -13.107 |
| **Bulgaria** | 0.812 | 0.812 | 0 | 269.013 | 0 | 3610.678 | 0 | -17.07 |
| **Croatia** | 0.787 | 0.787 | 0.001 | 9243.627 | 0 | 200064.77 | 0.001 | -14.471 |
| **Cyprus** | 0.623 | 0.623 | 0.001 | 210.602 | 0 | 3156.343 | 0.002 | -14.148 |
| **Czechia** | 0.193 | 0.193 | 0.001 | 28.294 | 0.001 | 69.478 | 0.001 | -12.409 |
| **Denmark** | 0.895 | 0.895 | 0 | 13.764 | 0 | 92.965 | 0.001 | -16.065 |
| **Estonia** | 0.872 | 0.872 | 0 | 1131.557 | 0 | 21529.182 | 0.001 | -15.888 |
| **Finland** | 0.862 | 0.862 | 0 | 153.612 | 0 | 3711.782 | 0.001 | -16.803 |
| **France** | 0.374 | 0.374 | 0.001 | 80.255 | 0.001 | 763.116 | 0.004 | -13.319 |
| **Germany** | 0.03 | 0.03 | 0.005 | 288.169 | 0.002 | 1029.848 | 0.004 | -9.761 |
| **Greece** | 0.784 | 0.784 | 0.002 | 125.8 | 0.001 | 2626.892 | 0.005 | -12.279 |
| **Hungary** | 0.845 | 0.845 | 0 | 438.833 | 9.01E-05 | 6360.769 | 0.001 | -16.933 |
| **Iceland** | 0.334 | 0.334 | 0 | 173.779 | 0 | 1148.339 | 0.001 | -14.945 |
| **Ireland** | 0.586 | 0.586 | 0.001 | 15.529 | 0 | 88.362 | 0.002 | -14.2 |
| **Italy** | 0.799 | 0.799 | 0.001 | 375.457 | 0 | 6121.609 | 0.003 | -13.586 |
| **Latvia** | 0.712 | 0.712 | 0 | 646.329 | 0 | 7392.764 | 0.001 | -15.394 |
| **Lithuania** | 0.386 | 0.386 | 0.001 | 363.36 | 0.001 | 3873.232 | 0.004 | -12.548 |
| **Luxembourg** | 0.846 | 0.846 | 0 | 29.062 | 0 | 349.201 | 0.001 | -15.712 |
| **Malta** | 0.260 | 0.26 | 0.001 | 201.556 | 0.001 | 1830.762 | 0.003 | -12.9 |
| **Netherlands** | 0.738 | 0.738 | 0.001 | 22.025 | 0 | 151.567 | 0.002 | -14.634 |
| **Norway** | 0.839 | 0.839 | 0 | 186.799 | 0 | 4056.019 | 0.001 | -16.678 |
| **Poland** | 0.777 | 0.777 | 0 | 997.969 | 0 | 14934.775 | 0.001 | -16.069 |
| **Portugal** | 0.006 | 0.006 | 0.087 | 450.367 | 0.032 | 795.849 | 0.296 | -4.341 |
| **Romania** | 0.602 | 0.602 | 0 | 28.102 | 0 | 138.406 | 0.001 | -14.822 |
| **Slovakia** | 0.547 | 0.547 | 0.001 | 356.282 | 0.001 | 7231.064 | 0.002 | -13.965 |
| **Slovenia** | 0.798 | 0.798 | 0 | 1554.403 | 0 | 10553.461 | 0.002 | -14.872 |
| **Spain** | 0.597 | 0.597 | 0.001 | 13.128 | 0.001 | 48.065 | 0.002 | -12.528 |
| **Sweden** | 0.854 | 0.854 | 0 | 274.337 | 0 | 6302.735 | 0.001 | -15.859 |
| **United Kingdom** | 0.312 | 0.312 | 0.001 | 12.668 | 0 | 36.582 | 0.001 | -13.362 |

***Table S8:*** *Fit metrics for generalized ARIMA models with optimal parameters for the ‘Unclassified’ group.* *No model could be built for Malta.* *A good fit in green colour is considered to be stationary R-squared above 0.65, R-squared above 0.85, MAPE below 6 and MaxAPE below 15. Moderate fit in yellow colour includes R-squared between 0.4 and 0.65, R-squared between 0.6 and 0.84, MAPE between 7 and 20 and MaxAPE between 16 and 40. Poor fit in orange colour is indicated by R-squared less than 0.4, R-squared less than 0.6, MAPE greater than 20 and MaxAPE greater than 40.*

| **Model Statistics for the 'Unclassified' group with ARIMA(1,0,1) without outlier detection** | | | | | | | | |
| --- | --- | --- | --- | --- | --- | --- | --- | --- |
| **Country** | **Stationary R-squared** | **R-squared** | **RMSE** | **MAPE** | **MAE** | **MaxAPE** | **MaxAE** | **Normalized BIC** |
| **Austria** | 0.239 | 0.239 | 0.006 | 928.804 | 0.004 | 4481.070 | 0.006 | -9.128 |
| **Belgium** | 0.465 | 0.465 | 0.010 | 369034.522 | 0.005 | 2938248.497 | 0.031 | -8.848 |
| **Bulgaria** | 0.374 | 0.374 | 0.025 | 353.379 | 0.014 | 2184.597 | 0.068 | -6.986 |
| **Croatia** | 0.892 | 0.892 | 0.011 | 3668.999 | 0.007 | 80575.561 | 0.028 | -8.648 |
| **Cyprus** | 0.169 | 0.169 | 0.000 | 96.914 | 0.000 | 1125.377 | 0.001 | -14.724 |
| **Czechia** | 0.157 | 0.157 | 0.003 | 18.731 | 0.002 | 36.412 | 0.004 | -10.388 |
| **Denmark** | 0.829 | 0.829 | 0.003 | 11.328 | 0.002 | 50.905 | 0.008 | -11.225 |
| **Estonia** | 0.024 | 0.024 | 0.001 | 7459.753 | 0.001 | 40879.154 | 0.005 | -13.045 |
| **Finland** | 0.789 | 0.789 | 0.006 | 5.796 | 0.005 | 15.572 | 0.010 | -9.944 |
| **France** | 0.658 | 0.658 | 0.010 | 18889.954 | 0.005 | 246363.586 | 0.022 | -8.878 |
| **Germany** | 0.140 | 0.140 | 0.078 | 171.439 | 0.034 | 559.644 | 0.061 | -4.063 |
| **Greece** | 0.816 | 0.816 | 0.002 | 299.763 | 0.001 | 4039.909 | 0.006 | -12.264 |
| **Hungary** | 0.523 | 0.523 | 0.001 | 56.386 | 0.001 | 396.748 | 0.003 | -12.810 |
| **Iceland** | 0.716 | 0.716 | 0.005 | 15.347 | 0.003 | 89.171 | 0.010 | -10.228 |
| **Ireland** | 0.452 | 0.452 | 0.000 | 329.556 | 0.000 | 1562.925 | 0.000 | -17.063 |
| **Italy** | . | . | 0.000 | 0.000 | 0.000 | 0.000 | 0.000 | -54.997 |
| **Latvia** | 0.851 | 0.851 | 0.001 | 112.588 | 0.001 | 1288.869 | 0.003 | -13.666 |
| **Lithuania** | 0.734 | 0.734 | 0.001 | 17.562 | 0.000 | 65.943 | 0.002 | -13.674 |
| **Luxembourg** | 0.581 | 0.581 | 0.011 | 23049.227 | 0.006 | 264341.838 | 0.024 | -8.538 |
| **Netherlands** | 0.761 | 0.761 | 0.008 | 72.902 | 0.003 | 1066.987 | 0.028 | -9.160 |
| **Norway** | 0.793 | 0.793 | 0.023 | 8.917 | 0.015 | 61.537 | 0.067 | -7.183 |
| **Poland** | 0.008 | 0.008 | 0.018 | 8188236.482 | 0.014 | 114556166.530 | 0.029 | -7.472 |
| **Portugal** | 0.611 | 0.611 | 0.000 | 479.889 | 0.000 | 3407.821 | 0.001 | -15.834 |
| **Romania** | 0.557 | 0.557 | 0.000 | 14.128 | 0.000 | 33.586 | 0.000 | -16.960 |
| **Slovakia** | 0.612 | 0.612 | 0.001 | 62.022 | 0.000 | 270.058 | 0.001 | -14.716 |
| **Slovenia** | 0.558 | 0.558 | 0.040 | 13229.218 | 0.027 | 143742.235 | 0.130 | -6.016 |
| **Spain** | 0.483 | 0.483 | 0.000 | 29.984 | 0.000 | 93.749 | 0.000 | -18.250 |
| **Sweden** | 0.709 | 0.709 | 0.008 | 6.141 | 0.005 | 28.904 | 0.024 | -9.158 |
| **United Kingdom** | 0.209 | 0.209 | 0.002 | 43.562 | 0.001 | 131.534 | 0.002 | -12.070 |

***Table S9:*** *Forecast of the proportion in % for the ‘Access’ group with ARIMA(1,0,1) for all countries until 2030.*

| **Forecast of the 'Access' group with ARIMA(1,0,1)** | | | | | | | | | | | | | | | | | | | | | | | | | | | | | | |
| --- | --- | --- | --- | --- | --- | --- | --- | --- | --- | --- | --- | --- | --- | --- | --- | --- | --- | --- | --- | --- | --- | --- | --- | --- | --- | --- | --- | --- | --- | --- |
| **Year** | **Austria** | **Belgium** | **Bulgaria** | **Croatia** | **Cyprus** | **Czechia** | **Denmark** | **Estonia** | **Finland** | **France** | **Germany** | **Greece** | **Hungary** | **Iceland** | **Ireland** | **Italy** | **Latvia** | **Lithuania** | **Luxembourg** | **Malta** | **Netherlands** | **Norway** | **Poland** | **Portugal** | **Romania** | **Slovakia** | **Slovenia** | **Spain** | **Sweden** | **United Kingdom** |
| **2020** | . | . | . | . | . | . | . | . | . | . | . | . | . | . | . | . | . | . | . | . | . | . | . | . | . | . | . | . | . | 67.9% |
| **2021** | . | . | . | . | . | . | . | . | . | . | . | . | . | . | . | . | . | . | . | . | . | . | . | . | . | . | . | . | . | 67.7% |
| **2022** | . | . | . | . | . | . | . | . | . | . | . | . | . | . | . | . | . | . | . | . | . | . | . | . | . | . | . | . | 69.0% | 67.4% |
| **2023** | . | . | . | . | 52.2% | . | . | . | . | . | . | . | . | . | . | . | . | . | . | . | . | . | . | . | . | . | . | . | 69.7% | 67.2% |
| **2024** | 58.2% | 68.3% | 42.5% | 61.0% | 50.6% | 61.0% | 79.9% | 64.5% | 73.6% | 72.3% | 58.2% | 42.4% | 50.5% | 82.8% | 77.3% | 48.6% | 71.9% | 65.4% | 60.3% | 55.8% | 71.6% | 61.6% | 62.0% | 62.7% | 52.0% | 43.1% | 63.1% | 62.0% | 70.0% | 67.1% |
| **2025** | 61.5% | 67.9% | 43.1% | 61.1% | 50.9% | 60.7% | 79.7% | 64.8% | 73.2% | 72.1% | 58.5% | 42.7% | 50.8% | 83.0% | 76.6% | 47.4% | 72.1% | 65.9% | 59.3% | 53.8% | 71.4% | 61.7% | 62.7% | 61.0% | 52.6% | 43.7% | 63.4% | 62.1% | 70.1% | 66.9% |
| **2026** | 58.9% | 67.5% | 43.7% | 61.2% | 50.9% | 60.7% | 79.5% | 65.0% | 73.0% | 71.9% | 58.6% | 43.0% | 51.0% | 83.1% | 75.9% | 47.3% | 72.3% | 66.4% | 58.4% | 52.4% | 71.2% | 61.8% | 63.3% | 59.6% | 52.9% | 44.1% | 63.6% | 62.2% | 70.2% | 66.8% |
| **2027** | 61.0% | 67.2% | 44.3% | 61.3% | 50.9% | 60.6% | 79.3% | 65.2% | 72.7% | 71.7% | 58.6% | 43.2% | 51.2% | 83.3% | 75.3% | 47.3% | 72.5% | 66.8% | 57.8% | 51.5% | 71.0% | 62.0% | 63.8% | 58.6% | 53.1% | 44.6% | 63.7% | 62.3% | 70.2% | 66.7% |
| **2028** | 59.3% | 66.9% | 44.9% | 61.4% | 50.9% | 60.6% | 79.1% | 65.4% | 72.5% | 71.5% | 58.6% | 43.4% | 51.5% | 83.4% | 74.7% | 47.3% | 72.7% | 67.2% | 57.3% | 50.8% | 70.8% | 62.1% | 64.1% | 57.7% | 53.3% | 45.1% | 63.7% | 62.3% | 70.2% | 66.6% |
| **2029** | 60.7% | 66.6% | 45.4% | 61.4% | 50.9% | 60.6% | 79.0% | 65.6% | 72.3% | 71.4% | 58.6% | 43.6% | 51.7% | 83.5% | 74.1% | 47.3% | 72.9% | 67.6% | 56.8% | 50.3% | 70.7% | 62.2% | 64.4% | 57.0% | 53.3% | 45.5% | 63.8% | 62.3% | 70.2% | 66.6% |
| **2030** | 59.5% | 66.3% | 45.9% | 61.5% | 50.9% | 60.6% | 78.8% | 65.8% | 72.2% | 71.2% | 58.6% | 43.8% | 51.8% | 83.6% | 73.6% | 47.3% | 73.1% | 68.0% | 56.5% | 49.9% | 70.6% | 62.4% | 64.7% | 56.4% | 53.4% | 45.9% | 63.8% | 62.3% | 70.2% | 66.5% |
| For each model. forecasts start after the last non-missing in the range of the requested estimation period. and end at the last period for which non-missing values of all the predictors are available or at the end date of the requested forecast period. whichever is earlier. | | | | | | | | | | | | | | | | | | | | | | | | | | | | | | |

***Table S10:*** *Forecast of the proportion in % for the ‘Watch’ group with ARIMA(1,0,1) for all countries until 2030.*

| **Forecast of the 'Watch' group with ARIMA(1,0,1)** | | | | | | | | | | | | | | | | | | | | | | | | | | | | | | |
| --- | --- | --- | --- | --- | --- | --- | --- | --- | --- | --- | --- | --- | --- | --- | --- | --- | --- | --- | --- | --- | --- | --- | --- | --- | --- | --- | --- | --- | --- | --- |
| **Year** | **Austria** | **Belgium** | **Bulgaria** | **Croatia** | **Cyprus** | **Czechia** | **Denmark** | **Estonia** | **Finland** | **France** | **Germany** | **Greece** | **Hungary** | **Iceland** | **Ireland** | **Italy** | **Latvia** | **Lithuania** | **Luxembourg** | **Malta** | **Netherlands** | **Norway** | **Poland** | **Portugal** | **Romania** | **Slovakia** | **Slovenia** | **Spain** | **Sweden** | **United Kingdom** |
| **2020** | . | . | . | . | . | . | . | . | . | . | . | . | . | . | . | . | . | . | . | . | . | . | . | . | . | . | . | . | . | 31.7% |
| **2021** | . | . | . | . | . | . | . | . | . | . | . | . | . | . | . | . | . | . | . | . | . | . | . | . | . | . | . | . | . | 31.9% |
| **2022** | . | . | . | . | . | . | . | . | . | . | . | . | . | . | . | . | . | . | . | . | . | . | . | . | . | . | . | . | 23.3% | 32.1% |
| **2023** | . | . | . | . | 47.7% | . | . | . | . | . | . | . | . | . | . | . | . | . | . | . | . | . | . | . | . | . | . | . | 22.0% | 32.3% |
| **2024** | 39.4% | 31.6% | 57.4% | 38.6% | 49.2% | 38.1% | 17.0% | 35.3% | 19.9% | 27.0% | 38.1% | 56.6% | 49.4% | 13.5% | 22.2% | 50.7% | 27.9% | 34.7% | 39.5% | 43.9% | 28.1% | 15.1% | 38.7% | 38.8% | 47.8% | 56.5% | 26.8% | 37.1% | 21.4% | 32.4% |
| **2025** | 38.0% | 32.1% | 56.9% | 38.2% | 48.9% | 38.2% | 17.2% | 35.1% | 20.1% | 27.2% | 37.0% | 56.3% | 49.1% | 13.5% | 22.9% | 52.0% | 27.7% | 34.2% | 40.6% | 45.8% | 28.5% | 15.3% | 38.0% | 38.5% | 47.2% | 56.1% | 27.2% | 37.1% | 21.1% | 32.5% |
| **2026** | 38.2% | 32.5% | 56.4% | 37.9% | 49.0% | 38.3% | 17.4% | 34.9% | 20.3% | 27.4% | 36.5% | 56.0% | 48.9% | 13.4% | 23.5% | 52.3% | 27.5% | 33.7% | 41.4% | 47.2% | 28.6% | 15.4% | 37.4% | 38.8% | 46.8% | 55.6% | 27.5% | 37.0% | 21.0% | 32.6% |
| **2027** | 38.1% | 32.9% | 55.9% | 37.6% | 49.0% | 38.3% | 17.6% | 34.6% | 20.4% | 27.6% | 36.3% | 55.8% | 48.6% | 13.4% | 24.2% | 52.4% | 27.3% | 33.3% | 42.0% | 48.2% | 28.7% | 15.6% | 36.8% | 38.5% | 46.6% | 55.1% | 27.8% | 37.0% | 20.9% | 32.7% |
| **2028** | 38.2% | 33.3% | 55.4% | 37.3% | 49.0% | 38.3% | 17.8% | 34.4% | 20.4% | 27.8% | 36.2% | 55.6% | 48.4% | 13.4% | 24.7% | 52.4% | 27.1% | 32.9% | 42.4% | 48.9% | 28.7% | 15.7% | 36.3% | 38.8% | 46.5% | 54.7% | 28.1% | 37.0% | 20.9% | 32.8% |
| **2029** | 38.2% | 33.6% | 54.9% | 37.1% | 49.0% | 38.3% | 17.9% | 34.2% | 20.5% | 28.0% | 36.2% | 55.5% | 48.2% | 13.4% | 25.3% | 52.5% | 26.9% | 32.5% | 42.8% | 49.4% | 28.7% | 15.8% | 35.9% | 38.5% | 46.5% | 54.3% | 28.3% | 37.0% | 20.9% | 32.9% |
| **2030** | 38.2% | 33.9% | 54.4% | 36.9% | 49.0% | 38.3% | 18.1% | 34.0% | 20.5% | 28.2% | 36.2% | 55.3% | 48.0% | 13.4% | 25.8% | 52.5% | 26.8% | 32.2% | 43.1% | 49.8% | 28.7% | 15.9% | 35.6% | 38.8% | 46.4% | 53.9% | 28.6% | 37.0% | 20.9% | 32.9% |
| For each model. forecasts start after the last non-missing in the range of the requested estimation period. and end at the last period for which non-missing values of all the predictors are available or at the end date of the requested forecast period. whichever is earlier. | | | | | | | | | | | | | | | | | | | | | | | | | | | | | | |

***Table S11:*** *Forecast of the proportion in % for the ‘Reserve’ group with ARIMA(1,0,1) for all countries until 2030.*

| **Forecast of the 'Reserve' group with ARIMA(1,0,1)** | | | | | | | | | | | | | | | | | | | | | | | | | | | | | | |
| --- | --- | --- | --- | --- | --- | --- | --- | --- | --- | --- | --- | --- | --- | --- | --- | --- | --- | --- | --- | --- | --- | --- | --- | --- | --- | --- | --- | --- | --- | --- |
| **Year** | **Austria** | **Belgium** | **Bulgaria** | **Croatia** | **Cyprus** | **Czechia** | **Denmark** | **Estonia** | **Finland** | **France** | **Germany** | **Greece** | **Hungary** | **Iceland** | **Ireland** | **Italy** | **Latvia** | **Lithuania** | **Luxembourg** | **Malta** | **Netherlands** | **Norway** | **Poland** | **Portugal** | **Romania** | **Slovakia** | **Slovenia** | **Spain** | **Sweden** | **United Kingdom** |
| **2020** | . | . | . | . | . | . | . | . | . | . | . | . | . | . | . | . | . | . | . | . | . | . | . | . | . | . | . | . | . | 0.4% |
| **2021** | . | . | . | . | . | . | . | . | . | . | . | . | . | . | . | . | . | . | . | . | . | . | . | . | . | . | . | . | . | 0.4% |
| **2022** | . | . | . | . | . | . | . | . | . | . | . | . | . | . | . | . | . | . | . | . | . | . | . | . | . | . | . | . | 0.3% | 0.4% |
| **2023** | . | . | . | . | 0.3% | . | . | . | . | . | . | . | . | . | . | . | . | . | . | . | . | . | . | . | . | . | . | . | 0.3% | 0.4% |
| **2024** | 1.1% | 0.1% | 0.1% | 0.3% | 0.3% | 0.3% | 0.2% | 0.2% | 0.2% | 0.4% | 0.6% | 1.0% | 0.1% | 0.1% | 0.5% | 0.6% | 0.2% | 0.2% | 0.3% | 0.2% | 0.2% | 0.2% | 0.1% | 3.3% | 0.2% | 0.3% | 0.2% | 0.8% | 0.3% | 0.4% |
| **2025** | 1.1% | 0.1% | 0.1% | 0.3% | 0.3% | 0.3% | 0.2% | 0.2% | 0.2% | 0.4% | 0.5% | 1.0% | 0.1% | 0.1% | 0.4% | 0.6% | 0.2% | 0.2% | 0.3% | 0.2% | 0.2% | 0.2% | 0.1% | 3.0% | 0.2% | 0.3% | 0.2% | 0.8% | 0.3% | 0.4% |
| **2026** | 1.1% | 0.1% | 0.1% | 0.3% | 0.2% | 0.3% | 0.2% | 0.2% | 0.2% | 0.4% | 0.5% | 1.0% | 0.1% | 0.1% | 0.4% | 0.6% | 0.2% | 0.2% | 0.3% | 0.2% | 0.2% | 0.2% | 0.1% | 2.8% | 0.2% | 0.3% | 0.2% | 0.7% | 0.3% | 0.4% |
| **2027** | 1.1% | 0.1% | 0.1% | 0.3% | 0.2% | 0.3% | 0.2% | 0.2% | 0.1% | 0.3% | 0.5% | 1.0% | 0.1% | 0.1% | 0.4% | 0.6% | 0.2% | 0.2% | 0.3% | 0.2% | 0.2% | 0.2% | 0.1% | 2.6% | 0.2% | 0.3% | 0.2% | 0.7% | 0.3% | 0.3% |
| **2028** | 1.1% | 0.1% | 0.1% | 0.3% | 0.2% | 0.3% | 0.2% | 0.2% | 0.1% | 0.3% | 0.5% | 1.0% | 0.1% | 0.1% | 0.4% | 0.5% | 0.2% | 0.2% | 0.2% | 0.2% | 0.2% | 0.2% | 0.1% | 2.5% | 0.2% | 0.3% | 0.2% | 0.7% | 0.3% | 0.3% |
| **2029** | 1.1% | 0.1% | 0.1% | 0.3% | 0.2% | 0.3% | 0.2% | 0.2% | 0.1% | 0.3% | 0.5% | 1.0% | 0.1% | 0.1% | 0.4% | 0.5% | 0.2% | 0.2% | 0.2% | 0.2% | 0.2% | 0.2% | 0.1% | 2.4% | 0.2% | 0.3% | 0.2% | 0.7% | 0.3% | 0.3% |
| **2030** | 1.1% | 0.1% | 0.1% | 0.3% | 0.2% | 0.3% | 0.2% | 0.2% | 0.1% | 0.3% | 0.5% | 1.0% | 0.1% | 0.1% | 0.4% | 0.5% | 0.2% | 0.2% | 0.2% | 0.2% | 0.2% | 0.2% | 0.1% | 2.3% | 0.2% | 0.2% | 0.2% | 0.7% | 0.3% | 0.3% |
| For each model. forecasts start after the last non-missing in the range of the requested estimation period. and end at the last period for which non-missing values of all the predictors are available or at the end date of the requested forecast period. whichever is earlier. | | | | | | | | | | | | | | | | | | | | | | | | | | | | | | |

***Table S12:*** *Forecast of the proportion in % for the ‘Unclassified’ group with ARIMA(1,0,1) for all countries until 2030.*

| **Forecast of the 'Unclassified' group with ARIMA(1,0,1)** | | | | | | | | | | | | | | | | | | | | | | | | | | | | | |
| --- | --- | --- | --- | --- | --- | --- | --- | --- | --- | --- | --- | --- | --- | --- | --- | --- | --- | --- | --- | --- | --- | --- | --- | --- | --- | --- | --- | --- | --- |
| **Year** | **Austria** | **Belgium** | **Bulgaria** | **Croatia** | **Cyprus** | **Czechia** | **Denmark** | **Estonia** | **Finland** | **France** | **Germany** | **Greece** | **Hungary** | **Iceland** | **Ireland** | **Italy** | **Latvia** | **Lithuania** | **Luxembourg** | **Netherlands** | **Norway** | **Poland** | **Portugal** | **Romania** | **Slovakia** | **Slovenia** | **Spain** | **Sweden** | **United Kingdom** |
| **2020** | . | 0.6% | . | . | . | . | . | . | . | . | . | 0.1% | 0.1% | . | . | 0.0% | . | . | 0.4% | . | . | . | . | . | . | . | . | . | 0.3% |
| **2021** | . | 0.6% | . | . | . | . | . | . | . | . | . | 0.1% | 0.2% | . | . | 0.0% | . | 0.2% | 0.4% | . | . | . | . | . | . | . | . | . | 0.3% |
| **2022** | . | 0.6% | . | . | . | . | . | . | . | . | . | 0.1% | 0.2% | . | . | 0.0% | . | 0.2% | 0.5% | . | . | . | . | . | . | . | . | 7.4% | 0.3% |
| **2023** | . | 0.6% | . | . | 0.1% | . | . | . | . | . | . | 0.1% | 0.2% | . | . | 0.0% | . | 0.2% | 0.6% | . | . | . | . | . | . | . | . | 7.6% | 0.3% |
| **2024** | 0.5% | 0.6% | 0.5% | 0.2% | 0.1% | 0.8% | 2.9% | 0.0% | 6.2% | 0.2% | 3.2% | 0.1% | 0.2% | 3.9% | 0.0% | 0.0% | 0.0% | 0.2% | 0.6% | 0.0% | 23.8% | 1.2% | 0.0% | 0.1% | 0.0% | 7.8% | 0.0% | 7.7% | 0.3% |
| **2025** | 0.7% | 0.6% | 0.7% | 0.3% | 0.1% | 0.8% | 2.9% | 0.0% | 6.3% | 0.3% | 5.3% | 0.1% | 0.2% | 3.8% | 0.0% | 0.0% | 0.0% | 0.2% | 0.7% | 0.1% | 23.7% | 1.2% | 0.0% | 0.1% | 0.0% | 6.6% | 0.0% | 7.9% | 0.3% |
| **2026** | 0.7% | 0.6% | 0.9% | 0.5% | 0.1% | 0.8% | 2.9% | 0.0% | 6.4% | 0.4% | 5.0% | 0.1% | 0.2% | 3.7% | 0.0% | 0.0% | 0.0% | 0.2% | 0.8% | 0.1% | 23.5% | 1.2% | 0.0% | 0.1% | 0.1% | 5.9% | 0.0% | 8.0% | 0.3% |
| **2027** | 0.7% | 0.6% | 1.0% | 0.6% | 0.1% | 0.8% | 2.9% | 0.0% | 6.5% | 0.5% | 5.1% | 0.1% | 0.2% | 3.6% | 0.0% | 0.0% | 0.0% | 0.2% | 0.8% | 0.1% | 23.3% | 1.2% | 0.0% | 0.1% | 0.1% | 5.6% | 0.0% | 8.1% | 0.3% |
| **2028** | 0.7% | 0.6% | 1.2% | 0.8% | 0.1% | 0.8% | 2.9% | 0.0% | 6.6% | 0.6% | 5.0% | 0.2% | 0.2% | 3.5% | 0.0% | 0.0% | 0.0% | 0.2% | 0.9% | 0.1% | 23.2% | 1.2% | 0.0% | 0.1% | 0.1% | 5.3% | 0.0% | 8.1% | 0.3% |
| **2029** | 0.7% | 0.6% | 1.3% | 0.9% | 0.1% | 0.8% | 2.8% | 0.0% | 6.6% | 0.6% | 5.0% | 0.2% | 0.2% | 3.5% | 0.0% | 0.0% | 0.0% | 0.3% | 0.9% | 0.2% | 23.0% | 1.2% | 0.0% | 0.1% | 0.1% | 5.2% | 0.0% | 8.2% | 0.3% |
| **2030** | 0.7% | 0.6% | 1.4% | 1.1% | 0.1% | 0.8% | 2.8% | 0.0% | 6.7% | 0.7% | 5.0% | 0.2% | 0.2% | 3.4% | 0.0% | 0.0% | 0.1% | 0.3% | 1.0% | 0.2% | 22.9% | 1.2% | 0.0% | 0.1% | 0.1% | 5.2% | 0.0% | 8.3% | 0.3% |
| For each model. forecasts start after the last non-missing in the range of the requested estimation period. and end at the last period for which non-missing values of all the predictors are available or at the end date of the requested forecast period. whichever is earlier. | | | | | | | | | | | | | | | | | | | | | | | | | | | | | |
